# Structural insights into the mechanism and E2 specificity of the RBR E3 ubiquitin ligase HHARI

**DOI:** 10.1038/s41467-017-00272-6

**Published:** 2017-08-08

**Authors:** Lingmin Yuan, Zongyang Lv, James H. Atkison, Shaun K. Olsen

**Affiliations:** 1 Department of Biochemistry & Molecular Biology, Medical University of South Carolina, Charleston, SC 29425 USA; 2 Hollings Cancer Center, Medical University of South Carolina, Charleston, 29425 SC USA

## Abstract

RING-in-between-RING (RBR) ubiquitin (Ub) E3 ligases function with Ub E2s through a RING/HECT hybrid mechanism to conjugate Ub to target proteins. Here, we report the crystal structure of the RBR E3, HHARI, in complex with a UbcH7 ~ Ub thioester mimetic which reveals the molecular basis for the specificity of this cognate E2/RBR E3 pair. The structure also reveals mechanistically important conformational changes in the RING1 and UBA-like domains of HHARI that accompany UbcH7 ~ Ub binding and provides a molecular basis by which HHARI recruits E2 ~ Ub in an ‘open’ conformation. In addition to optimally functioning with an E2 that solely performs transthiolation, our data suggests that HHARI prevents spurious discharge of Ub from E2 to lysine residues by: (1) harboring structural elements that block E2 ~ Ub from adopting a ‘closed’ conformation and (2) participating in contacts to ubiquitin that promote an open E2 ~ Ub conformation.

## Abstract (web summary)

HHARI is a RING-in-between-RING (RBR) ubiquitin (Ub) E3 ligase. Here the authors present the crystal structure of HHARI with the UbcH7 ~ Ub thioester intermediate mimetic, which reveals that HHARI binds this E2 ~ Ub in an open conformation and explains the specificity of this cognate RBR E3/E2 pair.

## Introduction

The reversible posttranslational modification of proteins by ubiquitin serves as a fundamental regulatory process used by cells to control nearly every aspect of eukaryotic biology^1^. The wide-ranging processes controlled by ubiquitination include cell cycle control, DNA repair, signal transduction, and immunity, among others^2–5^. Ub conjugation elicits its effects by altering the stability, localization, intermolecular interactions, and activity of target proteins^1^ and requires the sequential interactions and activities of three enzymes (E1, E2, and E3)^6–8^.

Following ATP-dependent activation of Ub that results in the formation of an E1 ~ Ub thioester intermediate, E1 transfers Ub onto the catalytic cysteine of E2 in a process termed thioester transfer (or transthiolation). The resulting E2 ~ Ub intermediate interacts with members of three different families of Ub E3 ligases (RING, HECT, and RING-in-between-RING (RBR)) that catalyze Ub conjugation to target proteins by distinct mechanisms^9–11^. The catalytic mechanism of RING E3s involves interactions between a zinc-binding RING domain and the E2 ~ Ub thioester intermediate that facilitates nucleophilic attack of the E2 ~ Ub thioester by a lysine on the target protein directly^9–11^. This contrasts with the mechanism of HECT E3s, which are structurally unrelated to RING E3s. HECT E3s participate in a process analogous to E1-E2 thioester transfer in which the conserved N-lobe of the catalytic HECT domain recruits E2 ~ Ub and subsequently transfers Ub to a catalytic cysteine within the conserved C-lobe to form an E3 ~ Ub thioester linkage that then undergoes attack by the target protein lysine to form the isopeptide bond^9–11^.

RBR E3s are a distinct class of Ub E3 ligases that harbor three tandem zinc-binding domains termed RING1, in-between RING (IBR) and RING2 (collectively called an RBR domain)^12, 13^. Surprisingly, RBR E3s were recently discovered to function through a RING/HECT hybrid mechanism^14^ in which the RING1 domain initially recruits the E2 ~ Ub thioester intermediate, similar to canonical RING E3s. However, rather than facilitating Ub discharge from E2 ~ Ub onto target protein lysine residues directly like a canonical RING E3, E2 ~ Ub binding to the RING1 domain of RBR E2s is followed by thioester transfer of Ub to a catalytic cysteine residue in the RING2 domain similar to HECT E3s^14–21^. An interesting question that arises is how RBRs prevent Ub discharge to lysine residues upon E2 ~ Ub binding to the RING1 domain (as for canonical RING E3s) and ensure that Ub is instead transferred to the RING2 catalytic cysteine prior to catalysis of ubiquitination (as for HECT E3s). A recent study suggested that one way that HHARI achieves this is by specifically inhibiting E2 ~ Ub conformations that are primed for catalysis of ubiquitin discharge onto lysine residues^22^; however, the structural basis for this observation is unknown.

HHARI (or ARIH1) is a member of the Ariadne family of RBR E3 ligases, which also includes TRIAD1 (ARIH2), ANKIB1 (KIAA1386), and Cullin-9 (PARC)^12^. In addition to the RBR domain, HHARI harbors an unstructured N-terminal domain rich in glycine and acidic residues, a UBA-like domain, and a helical C-terminal Ariadne domain that is characteristic of Ariadne subfamily members^23^. Crystal structures of full-length HHARI determined in the absence of E2 revealed an autoinhibitory HHARI conformation in which the RING1 domain is positioned far away from the RING2 catalytic cysteine residue, which is covered by Ariadne domain. The precise mechanisms by which HHARI autoinhibition is relieved and the conformational changes required for E2-RBR E3 Ub thioester transfer are unknown; however, recent studies have shown that Ariadne subfamily members HHARI and TRIAD1 are activated through interaction with Cullin RING ligases (CRLs) in their neddylated state^24, 25^.

An additional area of interest pertains to the specificity of HHARI interactions and function with ubiquitin E2s. UbcH7 (UBE2L3) was initially identified as RING1-dependent high-affinity binding partner of HHARI^26^, and there is evidence that HHARI and UbcH7 function together in pharyngeal development^27^, suggesting that they constitute a biologically relevant cognate E2/RBR E3 pair. Furthermore, a systematic screen of thirty-four different ubiquitin E2s for activity with the Ariadne subfamily member Triad1 revealed that only UbcH7-induced Triad1 autoubiquitination^24^. This is consistent with in vitro autoubiquitination assays with the untagged RBR domain of HHARI, which revealed significantly higher levels of activity with UbcH7 compared to UbcH5c^14^. These results suggest that there is a high degree of specificity in the HHARI/UbcH7 interaction, however, the molecular basis governing this specificity is unknown.

Here, we report the crystal structure of HHARI in complex with a UbcH7 ~ Ub thioester mimetic. The structure reveals mechanistically important UbcH7 ~ Ub-induced conformational changes in the RING1 and UBA-like domains of HHARI that play a key role in determining the specificity of this E2/E3 pair, as well as in promoting recruitment of UbcH7 ~ Ub in the ‘open’ conformation. We identify a residue specific to UbcH7 (Lys96) that plays a major role in determining its activity with HHARI, and we find that introducing this residue into UbcH5b substantially increases its activity, identifying this residue as a key specificity determinant. Overall, our structural and biochemical studies indicate that HHARI ensures transfer of Ub from E2 to the RING2 catalytic cysteine as opposed to discharge from E2 directly to lysine residues upon RING1 binding in at least three ways: (1) by evolving a mechanism to specifically recruit an E2 that solely performs transthiolation (UbcH7), (2) by harboring a loop insertion in the RING1 domain (unique to RBR E3s) that is involved in determining specificity of HHARI for UbcH7 and is incompatible with the UbcH7 ~ Ub binding in the ‘closed’ conformation primed for Ub discharge to lysine residues, and (3) contacts between Ub and the UBA-like domain that promote recruitment of UbcH7 ~ Ub in the inactive ‘open’ conformation.

## Results

### Overall structure of the HHARI/UbcH7-Ub complex

Recent data are consistent with a mechanism for RBR Ub E3 ligase activity in which the RBR RING1 domain initially recruits the E2 ~ Ub thioester intermediate, followed by transfer of Ub from E2 to a catalytic cysteine residue within the RBR RING2 domain^14, 16–21^. In order to gain insights into the molecular basis by which the RBR Ub E3 ligase HHARI recruits the E2 ~ Ub thioester intermediate, we used an E2 ~ Ub thioester intermediate mimetic that is stabilized by isopeptide bond formation between a lysine residue that substitutes for the E2 catalytic cysteine, and the C-terminal glycine of Ub (denoted as E2-Ub throughout)^28, 29^. We focused our efforts on the ubiquitin E2, UbcH7, because biochemical and biological data show that HHARI and UbcH7 interact with high affinity and function biologically as a cognate E2/E3 pair^23, 26, 27^. Using a slight modification of previously published protocols^28, 29^, we were able to produce large quantities of pure UbcH7 ~ Ub thioester intermediate mimetic, which as expected, forms an apparent 1:1 monomeric complex with full-length recombinant HHARI as assessed by gel filtration (Supplementary Fig. 1a).

**Fig. 1:**
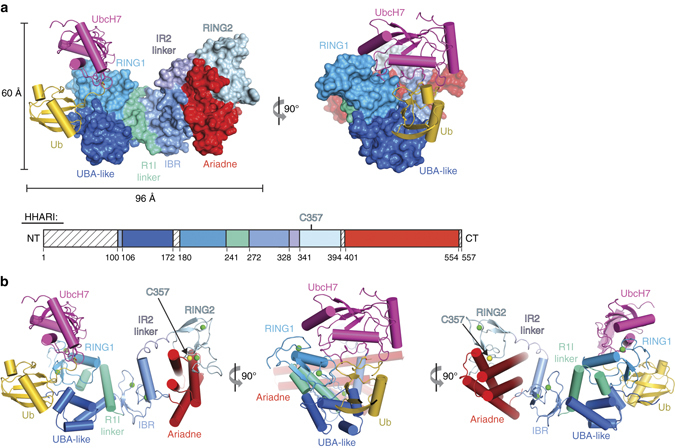
Overall structure of HHARI in complex with UbcH7-Ub. **a** HHARI is shown as a surface representation with individual domains labeled and color coded. UbcH7 (*magenta*) and Ub (*gold*) are shown as cartoons and the isopeptide bond linking C86K of UbcH7 to Gly76 of Ub is shown as *sticks*. A schematic outlining the domain organization of HHARI is shown on the *bottom* with regions of disorder in the structure indicated by *hatched boxes*. **b** Cartoon representation of the HHARI/UbcH7-Ub complex colored as in **a**. The isopeptide bond linking C86K of UbcH7 to Gly76 of Ub is shown as *sticks*. Zinc ions coordinated by the RING1, IBR, and RING2 domains of HHARI are shown as *green spheres* and the sulfur atom of the HHARI catalytic cysteine (Cys357) is shown as a *yellow sphere*

We determined the crystal structure of HHARI/UbcH7-Ub (Fig. 1a, b) to a resolution of 3.5 Å by molecular replacement using the structure of apo HHARI^23^ (HHARI^APO^; PDB: 4KBL) as the initial search model (Methods). Coordinates corresponding to UbcH7 and Ub were subsequently placed into unambiguous electron density evident after one round of refinement (Supplementary Fig. 1b–d). There is one HHARI/UbcH7-Ub complex per asymmetric unit and the final model was refined to *R*/*R*_free_ values of 0.226/0.252 (Table 1). Electron density corresponding to the isopeptide bond linking UbcH7 and Ub is evident in the structure (Supplementary Fig. 1c, d).

**Table 1:** Data collection and refinement statistics

	HHARI/UbcH7-Ub (PDB: 5TTE)
*Data collection*	
Space group	P6_5_22
Cell dimensions	
*a*, *b*, *c* (Å)	154.2, 154.2, 285.5
α, β, γ (°)	90, 90, 120
Resolution (Å)	49.7–3.50 (3.63–3.50)
*R*_merge_	0.094 (0.31)
*I* /*σI*	9.4 (1.3)
Completeness (%)	95.0 (82.0)
Redundancy	5.3 (2.6)
*Refinement*	
Resolution (Å)^a^	49.7–3.50 (3.59–3.50)
No. reflections	22,742
*R*_work_ / *R*_free_	0.226/0.252
No. atoms	
Protein	5545
Ligand/ion	6
Water	–
*B*-factors (Å^2^)	
Protein	120.9
Ligand/ion	149.1
Water	–
R.m.s. deviations	
Bond lengths (Å)	0.003
Bond angles (°)	0.520

^a^Values in parentheses are for highest-resolution shell

Overall, UbcH7-Ub-bound HHARI (HHARI^E2−Ub^) adopts an elongated architecture similar to that observed in HHARI^APO^ structures^23^ (PDB ID: 4KBL and 4KC9) with the RING1 and RING2 domains separated by ~ 90 Å at opposite ends of the structure and the catalytic cysteine residue of HHARI (Cys357) buried at the RING2/Ariadne domain interface (Fig. 1a, b). Almost all contacts between UbcH7 and HHARI involve the RING1 domain and the Ub molecule projects away from the surface of UbcH7, adopting an ‘open’ conformation that results in contacts to the UBA-like domain of HHARI (Fig. 1a, b and Supplementary Fig. 1e). That UbcH7 predominantly contacts the RING1 domain and that UbcH7-Ub is recruited to HHARI with Ub in the open conformation is fully consistent with previously published results^22, 23, 26^. The UbcH7 and HHARI catalytic cysteines residues are separated by ~ 54 Å, thus, the structure suggests that the UbcH7 ~ Ub intermediate can be recruited to HHARI in its autoinhibited conformation and that UbcH7-Ub binding does not induce large-scale conformational changes required for thioester transfer of Ub from UbcH7 to HHARI.

### HHARI conformational changes induced upon UbcH7-Ub binding

**Fig. 2:**
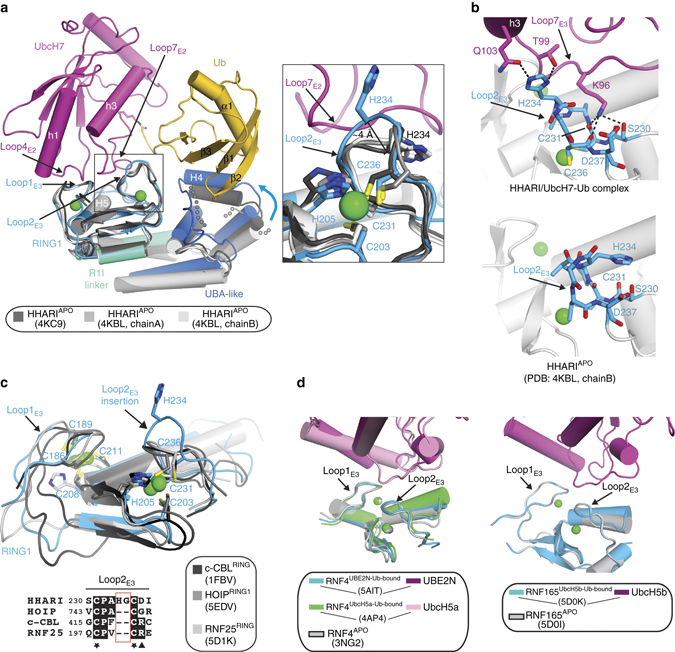
HHARI conformational changes accompany UbcH7-Ub binding. **a** Comparison of HHARI structures in the apo and UbcH7-Ub-bound states. The RING1 domains of the structures were superimposed. The HHARI/UbcH7-Ub structure is colored as in Fig. 1b and the three available apo HHARI structures are colored the indicated shade of *gray*. The RING1 Loop2_E3_ region of HHARI is boxed and a magnified cartoon representation of this region is presented as an *inset* to the *right*. The distance between the Cα atoms of His234 from Loop2_E3_ of apo and UbcH7-Ub bound HHARI structures is shown. Disordered regions in the UBA-like domains of apo HHARI that become ordered upon UbcH7-Ub binding are shown as semitransparent *gray spheres*. **b** The HHARI/UbcH7-Ub (*top*) and HHARI^APO^ (*bottom*) structures are shown as cartoon representations in the same orientation with selected residues shown as *sticks*. **c** Comparison of the HHARI RING1 domain to the RING domains of HOIP, c-Cbl, and RNF25. The Cα atoms of the eight residues involved in coordination of two zinc ions (*green spheres*) were superimposed and the structures are shown as cartoon representations with selected side chains shown as *sticks*. A sequence alignment of the Loop2_E3_ region of the RING domains is shown at the *bottom* with the loop insertion of HHARI boxed in *red* and highly conserved residues colored *black*. Cysteine residues involved in zinc coordination are indicated with a *black star* and the ‘linchpin’ arginine residue of canonical RING E3s that is involved in stabilization of the closed E2 ~ Ub conformation is indicated with a *black triangle*. **d** Comparison of the apo and E2-Ub bound crystal structures of RNF4 and RNF165. The structures are shown as cartoon representations the RING domains were superimposed to create the image

While large-scale conformational changes are not observed when comparing HHARI^APO^ and HHARI^E2−Ub^ structures, there are several subtle, but mechanistically important conformational changes that are observed in HHARI upon UbcH7-Ub binding (Fig. 2a). A large fraction of the contacts between the HHARI RING1 domain and UbcH7 involves a loop in the RING1 domain (termed Loop2_E3_), which is proximal to one of the two zinc-binding sites characteristic of the RING fold. While the Loop2_E3_ region adopts the same conformation in all HHARI^APO^ structures, comparison of HHARI^APO^ and HHARI^E2−Ub^ structures reveals a striking conformational change in the Loop2_E3_ region that is centered around His234 and Gly235 (Fig. 2a and Supplementary Fig. 2a–c). This Loop2_E3_ conformational change unmasks an E2-interacting surface that is buried in HHARI^APO^ structures and results in positioning of the loop closer to the surface of UbcH7 in the HHARI/UbcH7-Ub structure to facilitate additional intermolecular contacts (Fig. 2b). As noted previously^13, 22^, the RING1 domains of many RBR E3 ligases (including HHARI) are unique compared to canonical RING domains in that they have a one to six residue insertion in the Loop2_E3_ region that is positioned between two conserved residues involved in zinc coordination (Fig. 2c and Supplementary Fig. 2d–f). Interestingly, the aforementioned Loop2_E3_ conformational change observed in HHARI upon UbcH7 binding involves precisely the two residues comprising the HHARI Loop2_E3_ insertion (His234 and Gly235; Fig. 2a–c). Importantly, the conformational change in Loop2_E3_ is unique to the HHARI/UbcH7-Ub interaction as comparison of the RING domain of other E3s in the apo- vs. E2-bound states reveals Loop2_E3_ conformations that are essentially identical (Fig. 2d). The role that the RING1 Loop2_E3_ insertion and conformational change plays in HHARI mechanism will be discussed in further detail below.

Comparison of the HHARI^APO^ and HHARI^E2−Ub^ structures reveals additional HHARI conformational changes within the UBA-like domain. Relative to HHARI^APO^ structures, the UBA-like domain undergoes a rigid body rotation towards the UbcH7 ~ Ub thioester intermediate mimetic that facilitates contacts to Ub (Fig. 2a). The majority of the ubiquitin-interacting region of the UBA-like domain involves helix 4 (H4), which is immediately C-terminal to the three-helices comprising the canonical UBA domain fold, and a loop region immediately after H4. H4 is disordered in two of three HHARI^APO^ structures and the loop following the helix is disordered in all HHARI^APO^ structures and likely becomes ordered in the HHARI/UbcH7-Ub structure due to contacts to Ub (see details below).

### The HHARI/UbcH7 interface

**Fig. 3:**
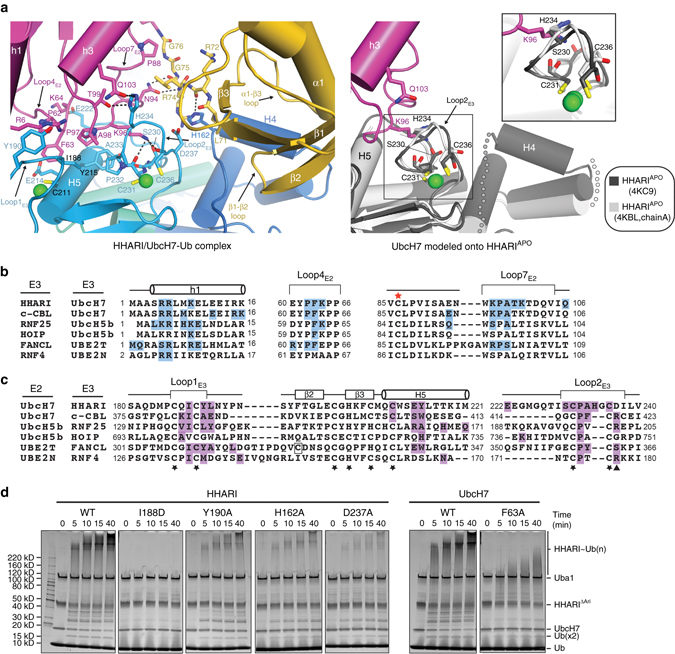
The HHARI^RING1^/UbcH7-Ub interface. **a** (*left*) Cartoon representation of the HHARI^RING1^/UbcH7-Ub interface with residues involved in intermolecular interactions shown as sticks. Zinc ions are shown as *green spheres* and hydrogen bonds are shown as *dashed lines*. (*right*) Model of an apo HHARI/UbcH7 complex created by superimposing RING1/UbcH7 from the HHARI/UbcH7-Ub structure onto RING1 of apo HHARI structures. Disordered regions in the UBA-like domains of apo HHARI that become ordered upon UbcH7-Ub binding are shown as semitransparent *gray spheres*. A magnified view of The HHARI Loop2_E3_ region is boxed and a magnified view is shown in the *inset*. **b** Structure-based sequence alignment of RING-interacting regions of selected ubiquitin E2s. E2 residues involved in contacts to the indicated RING domain are colored *blue*, the catalytic cysteines are indicated with a *red star*, and *dashes* indicate gaps in the alignment. Structures used to prepare the figure are c-CBL/UbcH7 (PDB:1FBV), RNF25/UbcH5b (PDB:5D1L), HOIP/UbcH5c (PDB:5EDV), FANCL/UBE2T (PDB:4CCG), and RNF4/Ube2N (PDB:5AIT). **c** Structure-based sequence alignment of ubiquitin E2-interacting regions of selected RING domains. RING domain residues involved in contacts to the indicated ubiquitin E2 are colored *magenta*, and the conserved zinc coordinating residues are indicated with *black stars*. The ‘linchpin’ arginine residue of canonical RING E3s is indicated with a *black* *triangle*. An atypical residue involved in zinc atom coordination (Cys324 of FANCL) is highlighted with a *black box* in the alignment. Structures used to prepare the alignment are the same as in **b**. **d** Structure-function analysis of the HHARI^RING1^/UbcH7-Ub interface. WT and mutant proteins were utilized in HHARI autoubiquitination assays for the indicated time points, as described in the Methods section. Full-length HHARI is catalytically inactive due to Ariadne domain-mediated autoinhibition so a catalytically active HHARI construct lacking the Ariadne domain (HHARI^ΔAri^) was used in these assays, as previously described^23^

A majority of the contacts between HHARI and UbcH7 involve a surface on the RING1 domain formed by two loops termed Loop1_E3_ and Loop2_E3_ that are positioned on either side of the domain, and an extended α helix (Helix 5, H5; Fig. 3a). Two loops, termed Loop4_E2_ and Loop7_E2_, and helix 1 (h1) of UbcH7 engage in the majority of contacts to the HHARI RING1 domain. Specifically, Loop4_E2_ contacts H5 and Loop1_E3_ of the RING1 domain; Loop7_E2_ engages in an extensive network of contacts with Loop2_E3_; and h1 of UbcH7 is perched above Loop1_E3_. Altogether, a total of ~ 1500 Å^2^ of surface area are buried at the HHARI RING1/UbcH7 interface with a mix of hydrophobic and hydrogen bond-mediated interactions.

At the center of the RING1/UbcH7 interface, Phe63, Pro97, and Ala98 of UbcH7 engage a hydrophobic patch on the RING1 domain formed by HHARI residues Ile188, Cys211, Glu214, and Tyr215 (Fig. 3a, left). This constitutes the conserved network of interactions present in most canonical RING E3/E2 structures determined to date (Fig. 3b, c and Supplementary Fig. 3a–c). As expected, mutations designed to disrupt this interaction, including an I188D HHARI mutation and a F63A UbcH7 mutation, significantly diminish HHARI activity as assessed by a previously established HHARI autoubiquitination assay that circumvents HHARI autoinhibition through deletion of the autoinhibitory Ariadne domain^23^ (Fig. 3d and Supplementary Fig. 3d). On the Loop1_E3_ side of the RING1 domain, Tyr190 of HHARI engages in a series of van der Waals contacts with the aliphatic portions of Arg6 and Lys9 on h1 and Lys100 within Loop7_E2_ of UbcH7 (Fig. 3a). The significant loss of activity resulting from a Y190A substitution in HHARI demonstrates importance of this set of interactions for normal HHARI function (Fig. 3d).

Contacts more specific to the HHARI/UbcH7 complex take place on the other side of the RING1 domain, where the E2-induced conformational change in Loop2_E3_ facilitates an extensive set of unique intermolecular interactions with UbcH7 (Fig. 3a, left). Most prominently, Lys96 of UbcH7 projects towards an acidic surface on HHARI that is unmasked as a result of Loop2_E3_ flipping upward towards the surface of the E2 compared to its conformation in HHARI^APO^ structures (Figs. 2b, 3a and Supplementary Fig. 4a). Lys96 of UbcH7 engages in a network of hydrogen bonds with this unmasked region, including the side chain of Ser230 and the backbone carbonyl oxygens of Cys231 and Cys236 (which are also involved in HHARI RING1 domain zinc ion coordination) (Figs. 2b, 3a). Lys96 is also positioned to engage in longer-range electrostatic interactions with Asp237 of HHARI Loop2_E3_. The importance of Asp237 to HHARI function is demonstrated by the significant loss of autoubiquitination activity observed with a D237A mutant (Fig. 3d), though surprisingly a D237R mutant behaved similar to wild type suggesting that electrostatic interactions specifically between the D237 side chain and UbcH7 K96 do not play an important role in activity (Supplementary Fig. 5d). Notably, this network of interactions is not compatible with the Loop2_E3_ conformation adopted in HHARI^APO^ structures, as Lys96 of UbcH7 would severely clash with the loop in its apo conformation (Fig. 3a, right and Supplementary Fig. 4b). An additional set of hydrogen bonds enabled by the Loop2_E3_ conformational change involves the side chains of His234 of HHARI and Thr99 and Gln103 of UbcH7.

To test the contribution of the length of Loop2_E3_ on HHARI functionality with UbcH7, we generated the following variants: (1) HHARIΔ^H234^ (2) HHARIΔ^H234/G235^, and (3) HHARI with a four residue (NTYS) insertion after G235 (HHARI^NTYS^). These four residues correspond to the sequence of HOIL, which has the longest Loop2_E3_ insertion among human RBR E3s. To test the contribution of Loop2_E3_ side chain composition, we generated the following variants: (1) HHARI^H234G^, (2) HHARI^H234A^, (3) HHARI^H234W^, and (4) HHARI^H234Q/G235D^ in which the HHARI ‘HG’ motif was mutated to the corresponding sequence of the Ariadne family member AriH2 (also known as Triad1). The general conclusion resulting from autoubiquitination assays with these proteins is that the length of the Loop2_E3_ insertion is important for HHARI activity, with both insertions and deletions significantly decreasing HHARI activity with UbcH7, whereas the side chain composition of Loop2_E3_ residues is less important (Supplementary Fig. 2g).

As mentioned previously, the RING1 domain of most RBR E3s differ from canonical RING domains in that they harbor a one to six residue insertion between the two zinc ion coordinating cysteine residues in Loop2_E3_ (Fig. 3c and Supplementary Fig. 2d). Interestingly, the region of HHARI Loop2_E3_ that clashes most severely with Lys96 of UbcH7 in the ‘apo’ state is the two-residue His234-Gly235 insertion (Fig. 3a, right and Supplementary Fig. 4b). Together, these structural observations suggest that the HHARI Loop2_E3_ conformational change induced upon UbcH7 binding is required for the HHARI/UbcH7 interaction to take place, and by extension, that a similar conformational change might be required in order for UbcH7 to function with other RBR E3s harboring a Loop2_E3_ insertion.

### Specificity in HHARI/ubiquitin E2 interactions

**Fig. 4:**
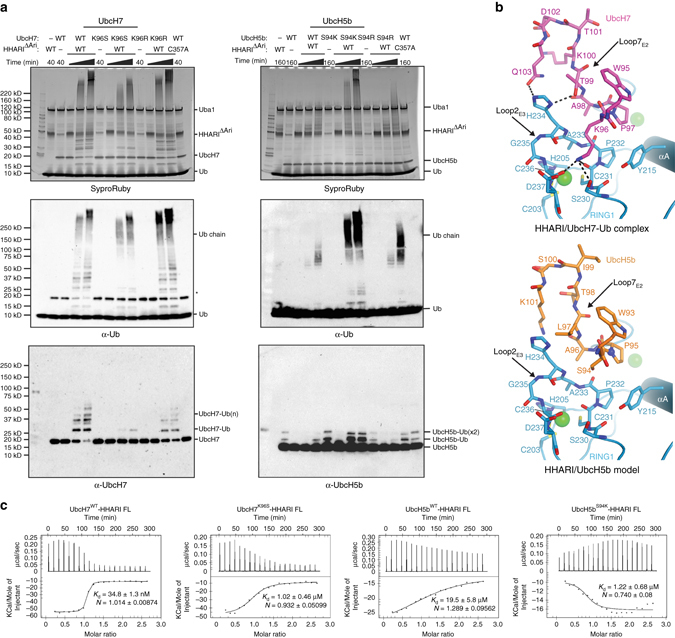
Specificity in HHARI/ubiquitin E2 interactions. **a** HHARI autoubiquitination assays. The indicated UbcH7 and UbcH5b variants were utilized in HHARI autoubiquitination assays for the indicated time points, as described in the Methods section. Time points for the timecourse experiment (represented by an extended *black triangle*) with UbcH7 variants were 0, 10, and 40 min. Time points for UbcH5b variants were extended to 0, 40, and 160 min to highlight the gain of function resulting from the S94K mutation relative to wild type UbcH5b. Gels were subjected to Sypro Ruby staining and α-Ub, α-UbcH7, and α-UbcH5b western blot as indicated. The *asterisk* indicates apparent cross-reactivity of the anti-ubiquitin antibody with UbcH7. **b** Intermolecular interactions at the Loop2_E3_/Loop7_E2_ region of the HHARI/UbcH7-Ub structure (*top*) and a model of HHARI in complex with UbcH5 (*bottom*). Zinc ions coordinated by the HHARI^RING1^ domain are shown as *green spheres* and hydrogen bonds are indicated with *black dashed lines*. **c** Isothermal titration calorimetry data for interactions between HHARI full-length and UbcH7^WT^, UbcH7^K96S^, UbcH5b^WT^, and UbcH5b^S94K^. *Upper* panels show raw power data and *lower* panels show fits of the data to standard binding equations using NanoAnalyze software (TA instruments)

HHARI and UbcH7 function together biologically^27^ and are defined as a cognate E3/E2 pair due to the high affinity with which they interact^23, 26^ and the fact that UbcH7 lacks intrinsic reactivity with lysine but can efficiently transfer Ub to cysteine^14^, a functionality that is a unique requirement of HECT and RBR E3 ligases. Although the highly promiscuous ubiquitin E2, UbcH5b, exhibits some degree of activity with HHARI in our autoubiquitination assay, UbcH7 exhibits a much higher level of activity (Fig. 4a). This comparatively high degree of activity does not appear to be a result of UbcH7 autoubiquitination as only low molecular weight UbcH7-Ub species are evident even at the longest time points of the assay (Fig. 4a). In order to gain insights into the apparent preference of HHARI for UbcH7 compared to UbcH5b, we performed a comparative analysis of residues at the HHARI/E2 interface via structure-based sequence alignment and by creating a HHARI/UbcH5b model (Fig. 4b and Supplementary Fig. 4c). The results of this analysis reveal that one of the most prominent differences between UbcH7 and UbcH5b is Ser94 of UbcH5b, which corresponds to Lys96 of UbcH7. As noted above, Lys96 of UbcH7 engages in an intricate network of interactions with HHARI that are facilitated by Loop2_E3_ conformational change that unmasks the UbcH7 interacting surface, whereas the HHARI/UbcH5b model shows that Ser94 cannot engage in an equivalent set of interactions due to its shorter side chain and lack of basicity (Fig. 4b).

To investigate the potential role that UbcH7 Lys96 and UbcH5b Ser94 play in the differential ability of these ubiquitin E2s to function with HHARI, we generated E2 mutants in which these residues were swapped. K96S UbcH7 activity is diminished compared to WT (Fig. 4a), underscoring the importance of the network of interactions involving Lys96 for UbcH7 functionality with HHARI. Consistent with our structural analysis, the S94K UbcH5b mutant exhibits a striking gain of function with HHARI compared to WT (Fig. 4a). The UbcH5b gain of function does not appear to be due to UbcH5b ubiquitination as only low molecular weight UbcH5b-Ub adducts are observed in these assays. Consistent with our biochemical data, a K96S substitution in UbcH7 decreases its affinity for full-length HHARI by ~ 30-fold (WT = 35 nM, K96S = 1020 nM *K*_d_), whereas a S94K substitution in UbcH5b increases its affinity for HHARI by ~ 16-fold (WT = 19.5 μM S94K = 1.2 μM *K*_d_) as assessed using isothermal titration calorimetry (Fig. 4c).

We subjected UBE2T, which harbors an arginine at the position corresponding to K96 of UbcH7 to the same assay and found that WT UBE2T exhibited a very-low level of activity with HHARI and that an R99K variant of UBE2T had a significantly higher level of activity (though still very low compared to UbcH7^WT^ and UbcH5b^S94K^ (Supplementary Fig. 4d). It is also worth noting that Ube2L6, which was identified along with UbcH7 as a high-affinity binding partner of HHARI via pulldown assays, also harbors a lysine residue at the position corresponding to Lys96 of UbcH7 (also Lys96). As expected we found that UBE2L6 interacts with HHARI as assessed by analytical gel filtration chromatography (Supplementary Fig. 4e), however, autoubiquitination assays show that UBE2L6 fails to exhibit robust levels of activity despite the presence of K96 (Supplementary Fig. 4d). Furthermore, in contrast to WT UbcH7, the comparatively low level of UBE2L6 activity was not further attenuated with a K96S UBE2L6 substitution (Supplementary Fig. 4d). UBE2S is another ubiquitin E2 that we found interacts with HHARI by gel filtration but has very little activity with HHARI in autoubiquitination assays (Supplementary Figs. 4d, e). E1–E2 thioester transfer assays indicate that the ability of the mutant E2 proteins used throughout this study to be charged with Ub is not diminished relative to WT (Supplementary Fig. 4f).

Overall, the data suggest that the presence of a lysine residue at the position corresponding to K96 of UbcH7 has a positive effect on E2 functionality with HHARI but that an intricate combination of factors collaborate to mediate high levels of activity. Since a single S94K substitution of UbcH5b unlocks relatively high levels of activity, the framework of residues outside of S94 are close to ‘ideal’. On the other hand, the very modest gain of function when a lysine residue is introduced into UBE2T suggests that the framework of residues outside of the position of interest that are required for high activity with HHARI are significantly less than ‘ideal’. Another point worth noting is that while a UbcH7^K96R^ variant retains very high levels of activity, this is not the case for UbcH5b^S94R^ (relative to UbcH5b^S94K^; Fig. 4a) nor is it the case for Ube2T which as noted harbors an arginine at the relevant position (Supplementary Fig. 4d). This suggests that there are very subtle differences in how these E2s are interacting with the RING1 domain that have a significant effect on activity. Finally, it is worth noting that there is not a direct correlation between the ability of ubiquitin E2s to interact with HHARI as assessed by gel filtration chromatography and the ability to function with HHARI in autoubiquitination assays. This is not surprising since after binding to the RING1 domain, the ubiquitin E2 must transfer Ub to the RING2 catalytic cysteine, a process that likely involves E2/RING2 contacts (see below). It is possible that the ubiquitin E2s we tested that bind with high affinity to HHARI but do not harbor activity in autoubiquitination assays exhibit this behavior due to a suboptimal ability to interact and function with the HHARI RING2 domain.

### Basis for recruitment of E2 ~ Ub in the open conformation

E2 ~ Ub thioester intermediates are known to adopt conformations ranging from ‘closed’ in which the Ub thioester folds back towards the surface of the E2 and engages in a network of interactions centered around helix 3 of the E2 (also known as the crossover helix), to ‘open’ and ‘backbent’ conformations in which Ub thioester extends away from this surface of the E2^30–32^. A series of recent studies has revealed that the mechanism by which canonical RING E3s catalyze isopeptide bond formation is through promotion of closed E2 ~ Ub conformations that optimally position the Ub C-terminus and E2 active site for catalysis^29, 33–36^.

An interesting apparent contradiction of HHARI biochemical behavior is that despite harboring a RING1 domain that recruits E2 in a manner resembling the RING domain of canonical RING E3s, the HHARI RING1/E2 interaction: (1) does not enhance reactivity of E2 ~ Ub toward free lysine, as assessed by lysine discharge assays^14^ and (2) is incapable of functioning with full-length HHARI in autoubiquitination^23^ (Fig. 4a) and CRL monoubiquitination assays^25^, as evidenced by a complete lack of activity when the RING2 domain catalytic cysteine (Cys357) is mutated to serine. A recent study revealed that these observations can at least partially be explained by HHARI failing to induce the E2 ~ Ub closed conformation and instead promoting recruitment of E2 ~ Ub in the catalytically inactive open conformation^22^.

**Fig. 5:**
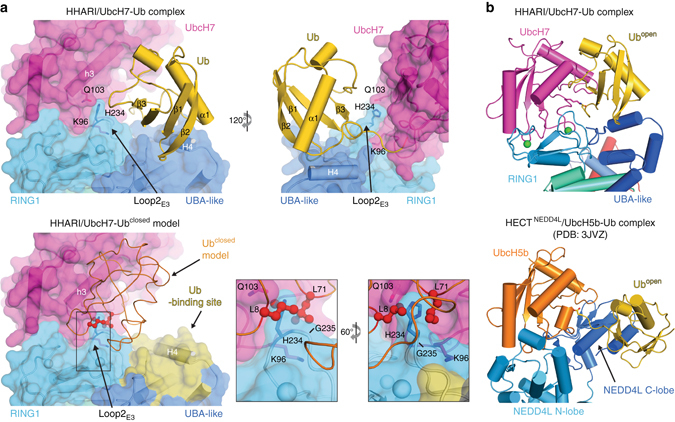
Molecular basis for HHARI recruitment of E2 ~ Ub in the open conformation. **a** (*top*) HHARI and UbcH7 from the HHARI/UbcH7-Ub structure are shown as cartoon representations with semitransparent surfaces. Ub is shown as a cartoon representation. Selected residues are shown as sticks. (*bottom*) A HHARI/UbcH7-Ub model with UbcH7-Ub in the ‘closed’ conformation shown in the same orientation as the *top* panel. The model was prepared by superimposing UbcH5b-Ub in the closed conformation (*orange*) (PDB: 4V3K) onto UbcH7 from the HHARI/UbcH7-Ub structure. The surface of the HHARI UBA-like domain that interacts with the core of Ub from the HHARI/UbcH7-Ub structure is colored *yellow*. Loop2_E3_ of HHARI is boxed and two magnified views of this region are shown in the *insets*. The two Ub residues that clash most severely with HHARI Loop2_E3_ in the closed conformation are colored *red* and shown as *ball* and *sticks*. **b** Comparison of the HHARI/UbcH7-Ub (left) and HECT^NEDD4L^/UbcH5b-Ub (*right*) complexes. Structures are shown as cartoon representations in the same orientation with proteins and domains labeled and color-coded

Analysis of the HHARI/UbcH7-Ub structure reveals the molecular basis for HHARI selectivity of UbcH7-Ub in the open conformation. First, as noted above, Ub projects away from the surface of UbcH7 in the complex and engages in a network of contacts with the HHARI UBA-like domain (Fig. 5a, b). Interestingly, the general architecture of the HHARI/UbcH7-Ub complex resembles that of the HECT^NEDD4L^/UbcH5b-Ub structure (Fig. 5b; PDB: 3JVZ)^37^. HECT E3s, like RBR E3s, must have mechanisms in place to prevent spurious discharge of Ub to lysine residues. In the HECT^NEDD4L^/UbcH5b-Ub structure Ub extends away from the UbcH5b active site and engages in contacts to the NEDD4L C-lobe akin to how Ub extends away from the UbcH7 active site and engages in contacts to the HHARI UBA-like domain in our structure (Fig. 5b). It is likely that these interactions serve as analogous mechanisms to promote the open E2-Ub conformation thereby preventing discharge to lysine residues.

**Fig. 6:**
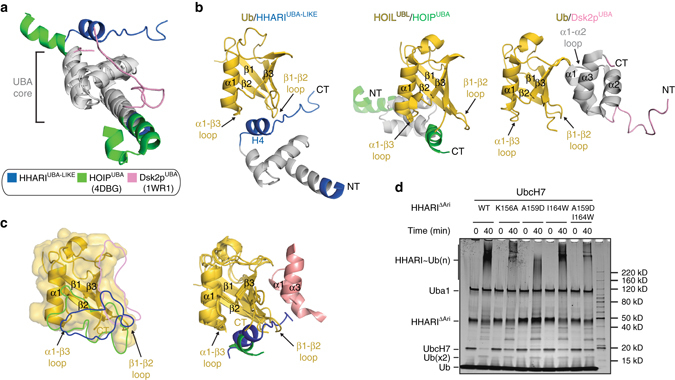
Analysis of the HHARI^UBA-like^/Ub interface. **a** Structural comparison of the UBA domains of HHARI, HOIP (PDB: 4DBG), and Dsk2p (PDB: 1WR1). The conserved three helix core of the UBA domains are colored *gray* and N- and C-terminal extensions unique to each protein are colored as indicated. **b** Comparison of the Ub/Ubl binding mode of the UBA domains of HHARI, HOIP, and Dsk2p. Ub/Ubl (*gold*) from the HHARI/UbcH7-Ub, HOIP^UBA^/HOIL1^UBL^ (PDB: 4DBG), and Ub/Dsk2p^UBA^ (PDB: 1WR1) structures were superimposed and are shown in the same orientation. **c** (*left*) The surfaces of Ub/Ubl that interact with the UBA-like domain of HHARI, HOIL, and Dsk2p are outlined on the surface of the Ubl domain from the HOIP^UBA^/HOIL^UBL^ structure. (*right)* The Ub/Ubl from the HHARI^UBA-like^/Ub, HOIP^UBA^/HOIL^UBL^, and Dsk2p^UBA^/Ub structures were superimposed and are shown as cartoon representations. The fragments of the UBA and UBA-like domains that contact the Ub/Ubl domains are shown as cartoon representations colored as in the *left* panel. **d** Structure-function analysis of the HHARI UBA-like/Ub interface. The indicated HHARI^ΔAri^ variants were subjected to autoubiquitination assays with UchH7 for the indicated time points, as described in the Methods section

The UBA-like/Ub interaction observed in the HHARI/UbcH7-Ub structure is atypical in that it does not involve contacts between the Ile44 hydrophobic patch of Ub and the α1-α2 loop and α3 helix of the UBA-like domain such as in the Dsk2/Ub interaction^38^ (PDB: 1WR1; Fig. 6a–c and Supplementary Fig. 5a). Rather, in the HHARI/UbcH7-Ub structure, the β1–β2 and α1–β3 loops of Ub straddle H4 of the UBA-like domain, which as mentioned above is outside (C-terminal) of the three helix bundle that defines the UBA domain. The β1–β2 loop of Ub is also wedged between H4 and a loop that immediately follows (Figs. 5a, and 6b, c). Importantly, this loop is disordered in all HHARI^APO^ structures (Fig. 2a) and presumably becomes ordered due to contacts to Ub in the HHARI/UbcH7-Ub structure. We found that introduction of K156A, A159D, I164W, and A159D/I164W mutations into the H4 region of the HHARI UBA-like domain resulted in a slight loss of activity in autoubiquitination assays (Fig. 6d).

Of all available structures of UBA/Ub complexes, it is interesting to note that the HHARI^UBA-like^/Ub interaction most closely resembles that of the HOIL-1L^UBL^/HOIP^UBA^ complex^39^ (PDB: 4DBG; Fig. 6b, c). In the HOIL-1L^UBL^/HOIP^UBA^ complex, the β1-β2 and α1-β3 loops of the Ubl domain straddles an α-helix that is C-terminal of the conserved three helix bundle of the UBA domain in a manner closely resembling the HHARI^UBA-like^/Ub interaction (Fig. 6a–c and Supplementary Fig. 5a). Although the positions on H4 involved in contacts to Ub/Ubl are conserved on the equivalent helix of HOIP^UBA^, the identity/similarity of these residues is poorly conserved (Supplementary Fig. 5b). Also, although the Ub/Ubl residues involved in contacts to the HHARI^UBA-like^ and HOIP^UBA^ domains reside on the same surface, the network of contacts is different, with hydrogen bonds predominating at the HHARI^UBA-like^/Ub interface and hydrophobic contacts predominating at the HOIP^UBA/^HOIL^UBL^ interface (Supplementary Fig. 5a). Overall, the binding modes of the HHARI^UBA-like^/Ub and HOIP^UBA/^HOIL^UBL^ complexes are superficially similar, with similar surfaces of the UBA-like/UBA domains interacting with similar surfaces of the Ub/Ubl, though the details of the interactions differ. Interestingly, the region of the HHARI UBA-like domain involved in contacts to Ub in our structure was recently identified as being important for HHARI+Neddylated-Cullin RING ligase (CRL)-mediated ubiquitination of a phosphopeptide substrate^25^. Surprisingly, we found that the binding affinity of UbcH7-Ub for HHARI (*K*_D_ = 272 nM) was lower than that of free UbcH7 (*K*_D_ = 35 nM) (Supplementary Fig. 4g), which we speculate might be the result of the tendency of UbcH7 ~ Ub to adopt the closed conformation^22^ that is incompatible with HHARI binding.

With regards to the flexible Ub C-terminus there are several notable contacts observed in the structure including His162 of HHARI, which is wedged between the side chains of Leu71 and Arg74 of Ub and engages in a hydrogen bond to the backbone oxygen of Arg72 (Fig. 3a). A HHARI H162A mutation significantly diminishes activity relative to WT, highlighting the importance of this residue (Fig. 3d). Closer to the covalent linkage between UbcH7 and Ub Asn94 of UbcH7 engages in a pair of hydrogen bonds with the backbone oxygen of Arg74 and Pro88 of UbcH7 engages in van der Waal contacts to Gly76 of Ub, the carbonyl oxygen of which projects towards the side chain of UbcH7 His119 (Fig. 3a).

While contacts between Ub, UbcH7, and the UBA-like domain are a means of promoting an open UbcH7-Ub conformation, there are also mechanisms in place that hinder UbcH7-Ub binding to HHARI in the closed conformation. We created a model of a HHARI/UbcH7-Ub(closed) using the structure of RNF38/UbcH5b-Ub^40^ (PDB: 4V3K) as a guide (Fig. 5a, *bottom*). The model reveals that the Loop2_E3_ insertion of the HHARI RING1 domain (His234-Gly235) sterically clashes with Ub in the closed conformation, in particular residues Leu8 and Leu71 (Fig. 5a, *bottom*). These clashes involve both main chain and side chain atoms of the Loop2_E3_ insertion of HHARI and take place whether the Loop2_E3_ insertion is in the conformation observed in all HHARI^APO^ structures or that observed in the HHARI/UbcH7-Ub structure. Since Loop2_E3_ is very closely positioned to Ub(closed) in all canonical RING E3s, it is likely that other RBR E3s with Loop2_E3_ insertions would select against closed E2-Ub conformations through a similar mechanism. It is also noteworthy that the surface of UbcH7 that interacts with Ub in the closed conformation is partially engaged by Loop2_E3_ of HHARI RING1, in particular the interaction between His234 of HHARI and Gln103 in helix 3 of UbcH7. Finally, the ‘linchpin’ arginine residue conserved within canonical RING domains that engages in contacts with Ub to stabilize the E2-Ub closed conformation is not conserved in RBR E3s (Fig. 3c and Supplementary Fig. 5c). This lack of sequence conservation appears to be a secondary factor in accounting for the lack of UbcH7’s lack of reactivity with HHARI RING1 because a D237R mutant containing the ‘linchpin’ arginine residue functions similar to wild type and a D237R/C357A double mutant lacking the RING2 domain catalytic cysteine residue harbors no apparent activity in HHARI autoubiquitination assays (Supplementary Fig. 5d).

### HHARI activation and comparision to other RBR E3s

**Fig. 7:**
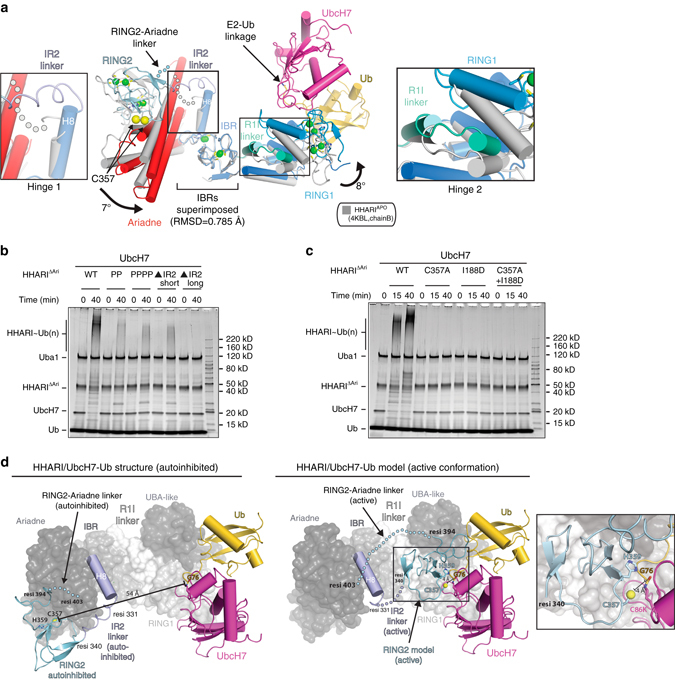
Modeling conformational changes accompanying thioester transfer of Ub from E2 to HHARI. **a** Comparison of apo and E2 bound HHARI reveal conformational changes in the IR2/RING2 region. The IBR domains of apo HHARI (PDB: 4KBL, chain B) and E2 bound HHARI were superimposed and the structures are shown as cartoon representations. The hinge 1 and hinge 2 regions are boxed and magnified views are shown to the *left* and *right* of the structure, respectively. *Bold black arrows* indicate the direction of the rotation resulting from conformational changes in hinges 1 and 2. Disordered linkers connecting the IBR and RING2 domain and the RING2 and Ariadne domains are shown as *semitransparent spheres*. **b** Structure-function analysis of the HHARI IR2 linker. WT, mutant HHARI^ΔAri^ proteins were used in HHARI autoubiquitination assays for the indicated time points, as described in the Methods. “PP” refers to T333P/S334P HHARI double mutant. “PPPP” refers to T333P/S334P/A338P/A339P HHARI quaternary mutant. “ΔIR2 short” and “ΔIR2 long” refer to Δ333–339 and Δ322–339 HHARI deletion mutants, respectively. **c** Evaluation of *cis* vs. *trans* mechanisms for HHARI activation. The indicated HHARI^ΔAri^ variants were subjected to autoubiquitination assays with UchH7 for the indicated time points, as described in the Methods. **d** (*left*) The HHARI/UbcH7-Ub structure with Ub(t) (*gold*), UbcH7 (*magenta*), and the HHARI IR2 linker (*slate*) and RING2 domain (*light blue*) shown as cartoon representations. Other HHARI domains are shown as a transparent surface representation. The disordered region linking the RING2 and Ariadne domains is shown as semitransparent *light blue spheres*. (*right*) A model of an ‘active’ HHARI/UbcH7-Ub complex was created using the structure of the HOIP/UbcH5b-Ub complex (PDB:5EDV) as a guide. The hypothetical conformational changes of the HHARI IR2 and RING2-Ariadne linkers required for the RING2 and UbcH7 domains to come in proximity during thioester transfer are indicated with semitransparent slate colored spheres. The RING2/UbcH7 interface is boxed with a magnified view shown in the *inset*

Analysis of our HHARI/UbcH7-Ub complex shows that HHARI^E2−Ub^ adopts a similarly elongated structure as HHARI^APO^ which indicates that E2 ~ Ub binding does not trigger conformational changes necessary for thioester transfer. However, the Cα atoms of the UbcH7 and HHARI catalytic cysteines are 8 Å closer compared to the HHARI^APO^ structure and superimposition of the IBR domains reveal conformational changes in two hinge regions that account for this difference and may provide insights to the larger structural changes that occur during thioester transfer (Supplementary Fig. 6a). The first hinge region is centered on Helix 8 within the IBR domain which rotates ~ 7 degrees towards E2 ~ Ub, and the second hinge region is centered on the linker that connects the RING1 and IBR domains (R1I linker), which rotates ~ 8 degrees toward the RING2 domain (Fig. 7a).

In addition to Hinges 1 and 2, an additional region that is likely to be involved in conformational changes required for the E2 and HHARI active sites to come in proximity during thioester transfer is the linker that connects the IBR and RING2 domains (IR2 linker). In all HHARI^APO^ structures, the IR2 linker is disordered and though this region is helical in the E2 bound state (Fig. 7a), the elevated B-factors of residues in this linker are consistent with a high degree of flexibility. As noted previously, the IR2 forms a short helix in the HHARI RING2 NMR structure^19^ and in the HOIP/UbcH5b-Ub structure^15^. The IR2 linker harbors many of the residues recently identified as being involved in binding to ubiquitin thioester by HHARI^22^ and HOIP,^15^ which led to the proposal that this region undergoes a coil-to-helix transition that effectively assembles the ubiquitin binding site, thereby facilitating thioester transfer of Ub to the active site cysteine of the RING2 domain. Our structure thus suggests that the coil-to-helix transition can occur upon UbcH7-Ub binding. However, since the Ub thioester is held in the open conformation through contacts with the UBA-like domain in our HHARI/UbcH7-Ub structure, additional conformational changes must occur for ubiquitin and the IR2 linker/RING2 to come into proximity during HHARI activation.

Interestingly, Neddylated-CRLs have recently been shown to interact with HHARI and Triad1 UBA-like domains via the Nedd8 molecule that triggers HHARI/Triad1 activation^24, 25^. It is tempting to speculate that Nedd8 binding to the UBA-like domain releases the interaction with ubiquitin which can then ‘swing around’ via its flexible C-terminus to participate in interactions with the IR2 linker/helix identified by Dove et al. for HHARI and Lechtenberg et al., for HOIP. In both the HHARI^APO^ and HHARI/UbcH7-Ub structures, first helix of the UBA-like domain blocks the pUb/Ub^allo^ binding sites important for Parkin^41–43^ and HOIP^15^ activity, respectively (Supplementary Fig. 6b, c). This implies that if an event analogous to pUb/Ub^allo^ binding occurs during HHARI activation, that the UBA-like domain must be displaced from it position in the HHARI/UbcH7-Ub structure. Whether Nedd8 binding to the HHARI UBA-like domain triggers conformational changes that free up the pUb/Ub^allo^ binding site from Parkin and HOIP structures that is sterically blocked by the first helix of the UBA-like domain of HHARI is unclear. Related to this, since pUb binding to Parkin has been shown to trigger conformational changes in the pUBH helix that results in IBR conformational changes that facilitate E3 activation^41^, it is also unclear whether a mechanism akin to pUBH/h_E2_ helix extension is conserved in HHARI. Considering that the first helix of UBA domains is typically involved in Ub/Ubl interactions, it is also possible that a canonical UBA-like/Nedd8 interaction mechanistically mimics the pUBH/h_E2_ interaction as they are located at similar regions of the structure.

The IR2 linker region of a symmetry related HOIP molecule engages in contacts to the Ub thioester in the HOIP/UbcH5b-Ub structure that involves the I44 hydrophobic patch of the Ub thioester^15^. As noted above, Dove et al. identified a potential interaction between Ub and the IR2 linker^22^, which is in principle consistent with the observations in the HOIP/UbcH5b-Ub structure. With that said, the set of key ubiquitin residues identified in the Dove et al. study that are involved in contacts to HOIP, Parkin, and HHARI were overlapping but distinct^22^, suggesting differences in the details of how the ubiquitin donor interacts with different RBR E3s. Furthermore, in order for Ub donor to fit onto a similar binding pocket as that observed in the HOIP/UbcH5b-Ub structure it would require distinct conformational changes in HHARI, as there are steric clashes between the Ub donor and elements of RING1 and the IBR (IBRs superimposed; Supplementary Fig. 6b).

Consistent with the idea that flexibility of the IR2 linker is important for HHARI activation, we found that introduction of proline residues and deletions in this region results in a significant reduction in HHARI activity (Fig. 7b). Furthermore, we found that mixing I188D HHARI, which is deficient in E2 binding with C357A HHARI which cannot be charged with Ub, resulted in no activity in HHARI autoubiquitination assays (Fig. 7c). Together with the IR2 mutant/truncations described above, the most likely explanation for these results is that recruitment of E2 ~ Ub and thioester transfer of Ub to the RING2 active site cysteine occurs in *cis*, and that conformational changes in the IR2 linker facilitate the E2 and E3 active sites coming into proximity during this process. Presumably conformational flexibility of the linker between RING2 and the Ariadne domain (residues 395–400), which is completely disordered in the HHARI/UbcH7-Ub and all HHARI^APO^ structures, is also required for liberation of the RING2 domain from the autoinhibitory Ariadne domain, which thereby frees the HHARI catalytic cysteine and results in its placement proximal to the UbcH7 ~ Ub thioester linkage (Fig. 7d).

## Discussion

The HHARI/UbcH7-Ub structure presented herein reveals how HHARI selects for E2 ~ Ub thioester intermediate in the open conformation and selects against interaction with E2 ~ Ub in the closed conformation, thereby providing a molecular basis for how HHARI restricts discharge of Ub from E2 to the catalytic cysteine on the RING2 domain rather than functioning like a canonical RING E3 that primes E2 ~ Ub for discharge of Ub to lysine residues. The Loop2_E3_ insertion of the RING1 domain is one of the major determinants of the steric selection against E2 ~ Ub recruitment in the closed conformation and the presence of this insertion in a majority of RBR E3 ligases suggests that this may be a relatively conserved mechanism for how this family of E3s restricts Ub discharge to the E3 catalytic cysteine. Our structural and biochemical experiments have identified an amino acid present in UbcH7 (Lys96) that is a major determinant of the specificity of the HHARI/UbcH7 ~ Ub interaction and we find that introduction of this amino acid into UbcH5b results in a conversion from comparatively low to high levels of activity with HHARI, potentially providing a platform to study the role of HHARI/E2 specificity in a cellular context. Finally, although HHARI remains in the autoinhibited conformation in the HHARI/UbcH7-Ub structure, a comparison to HHARI^APO^ structures reveals hinge regions that are likely to drive the conformational changes required for thioester transfer of Ub from E2 to the catalytic cysteine in the RING2 domain.

During revision of this manuscript, a related study describing a HHARI/UbcH7-Ub crystal structure was published^44^. As it pertains to the HHARI/UbcH7 interface and contacts important for molecular recognition, both studies resulted in the same major conclusions. Both studies also resulted in the same major conclusion with regards to the mechanism by which the HHARI Loop2_E3_ insertion prevents recruitment of UbcH7 ~ Ub in the closed conformation. There is one significant difference between the two papers with regards to the location of the Ub thioester mimetic in the complex. In our structure, the single HHARI/UbcH7-Ub complex in our asymmetric unit is a 1:1:1 complex in which the ubiquitin molecule is very well-ordered and interacts in the open conformation with the HHARI UBA-like domain in a non-canonical manner as described above. In the recently published Dove et al. study, only one of two Ub molecules in the asymmetric unit of their crystal is modeled, and rather than E2 and Ub interacting with the same HHARI molecule as a 1:1:1 complex, the Ub molecule is involved in crystal contacts with the UBA-like domain of a symmetry related HHARI molecule in a manner resembling a canonical UBA/Ub interaction^44^. The authors biochemically probed the canonical Ub/UBA-like interface observed in their crystal and did not observe an effect on HHARI autoubiquitination^44^, though it is formally possible that the observed interaction may occur with free Ub or Nedd8 from during bona fide activation of HHARI (such as through interactions with Neddylated-CRL). Elucidation of the precise architecture of the active HHARI/UbcH7 ~ Ub complex, including the potential role of UBA-like interactions with Ub/Nedd8 will be an exciting focus of future investigation that will require further characterization of the factors that trigger HHARI activation, such as interactions with neddylated CRLs^24, 25^.

## Methods

### Protein expression and purification

The DNA fragments encoding residues 1–557 (full-length) and 1–400 (∆Ariadne) of *HHARI*, respectively were cloned into vector pSMT3^45^ encoding for an N-terminal 6×His-SMT3 tag with a ULP1 cleavage site. For crystallization, *UbcH7* with the mutation C86K (to enable isopeptide linkage to ubiquitin) was cloned into pET28 vector with C-terminal uncleavable 6×His tag. For enzymatic assays, wild type (WT) *UbcH7* was cloned into the same vector. WT *UbcH5b* was cloned into pMTTH vector that codes for a C-terminal 6×His tag with a TEV cleavage site. Primer sequences used to generate all constructs in this study are listed in Supplementary Table 1.

All proteins were expressed in BL21 (DE3) Codon Plus *E.coli* (Agilent Technologies) after induction with 0.1 mM isopropyl-β-D-1-thiogalactoside (IPTG) overnight at 18 °C. For expression of HHARI constructs, 0.1 mM ZnCl_2_ was added before induction. Bacterial cultures were collected by centrifugation, and lysed by sonication in lysis buffer (20 mM Tris HCl pH 8.0, 350 mM NaCl, 20 mM Imidazole, 2 mM 2-Mercaptoethanol (βME)), in the presence of DNase. The suspension was centrifuged and the supernatant applied to Ni-NTA agarose (Qiagen). For HHARI constructs, 6×His-SMT3 tags were removed by addition of ULP1 protease at a ratio of 1:1000 (w/w) overnight at 4 °C. HHARI constructs were further purified using HiLoad 26/600 Superdex 200 pg size-exclusion chromatography column (GE Healthcare) equilibrated in buffer (20 mM Tris HCl pH 8.0, 350 mM NaCl, 2 mM βME) and MonoQ 10/100 GL (GE Healthcare) in 20 mM Tris HCl pH 8.0, 50 mM NaCl, 2 mM βME and eluted with a gradient from 50–1000 mM NaCl. For UbcH5b constructs, 6×His tags were removed by addition of TEV protease at a ratio of 1:100 (w/w) overnight at 4 °C. UbcH7 constructs and UbcH5b constructs were further purified on a HiLoad 26/600 Superdex 75 pg size-exclusion chromatography column (GE Healthcare) equilibrated in buffer (20 mM Tris HCl pH 8.0, 350 mM NaCl). UbcH7 constructs were further purified by using MonoS 10/100 GL (GE Healthcare) in 20 mM Bis-Tris pH 6.5, 50 mM NaCl and eluted with a gradient from 50–1000 mM NaCl.

### UbcH7-Ub conjugate

UbcH7-Ub conjugate was generated based on published methods^28, 29^ with some modification. Briefly, 0.5 μM Uba1, 5 μM UbcH7 and 25 μM Ub were mixed in a buffer containing 50 mM Tris HCl pH9.5, 50 mM NaCl, 2 mM ATP, 10 mM MgCl_2_, and 1 mM βME. The mixture was incubated at 35 °C for 16 h. The UbcH7-Ub conjugate was purified by using MonoS 10/100 GL (GE Healthcare) in 20 mM Bis-Tris pH 6.5, 50 mM NaCl and eluted with a gradient from 50–1000 mM NaCl and HiLoad 26/600 Superdex 75 pg size-exclusion chromatography column (GE Healthcare) equilibrated in buffer (20 mM Tris HCl pH 8.0, 100 mM NaCl).

### HHARI/UbcH7-Ub complex formation

HHARI was mixed with a 2-fold molar excess of UbcH7-Ub and incubated overnight at 4 °C. The mixture was then applied to a Superose 12 10/300 GL size-exclusion chromatography column equilibrated in 20 mM Tris HCl pH 8.0, 50 mM NaCl and 2 mM βME. Complex formation and purity was confirmed using SDS-PAGE, and complex containing fractions were pooled and concentrated to 10 mg ml^−1^ for crystallization.

### HHARI/UbcH7-Ub crystallization

Crystals were grown at 18 °C using the hanging drop vapor diffusion method by mixing 1 μl of protein complex with 1 μl reservoir solution (100 mM sodium cacodylate pH 6.17, 350 mM sodium acetate, 10% Glycerol). Crystals appeared after 3 days and grew to their final size within 7 days. Crystals were briefly soaked in a cryoprotectant solution (100 mM sodium cacodylate pH 6.17, 2 M sodium acetate, 20% Glycerol) before flash-freezing in liquid nitrogen.

### Structure determination and refinement

X-ray diffraction data for the HHARI/UbcH7-Ub complex was collected on a Pilatus 6MF detector at Advanced Photon Source (Argonne, Illinois, USA), NE-CAT beamline 24-ID-C. All data were indexed, integrated, and scaled using HKL2000^46^. The HHARI/UbcH7-Ub crystal belongs to space group P6_5_22 with unit cell dimensions *a* = 154.2, *b* = 154.2, *c* = 285.5. There is one HHARI/UbcH7-Ub complex per asymmetric unit.

A complete data set for the HHARI/UbcH7-Ub crystals was collected to a resolution of 3.5 Å. The program PHASER^47^ was used to find an initial molecular replacement solution using a multiple ensemble search comprising the UBA-RING1, IBR, and RING2-Ariadne fragments derived from a structure of HHARI determined in the absence of E2 (PDB: 4KBL). After one round of refinement the resulting maps were inspected and electron density for UbcH7 was evident. Coordinates for UbcH7 (PDB: 4Q5E) were manually placed into the electron density and following an additional round of refinement coordinates for ubiquitin (PDB: 4II2) were manually placed into unambiguous electron density. The model was refined to *R*/*R*_free_ values of 0.226/0.252 via iterative rounds of refinement and rebuilding using PHENIX^48^ and COOT^49^.

### Autoubiquitination assay

Autoubiquitination assays were performed in 20 mM HEPES pH 7.5, 50 mM NaCl, 2.5 mM MgCl_2_ and using 150 nM Uba1, 500 nM E2, 250 nM HHARI and 20 μM Ub. Reactions were initiated by addition of 2.5 mM ATP and were incubated at room temperature. Reaction samples were quenched at the indicated time points in SDS-PAGE loading buffer containing 10% βME, resolved on 4–15% gradient gels (Bio-Rad), and visualized by SYPRO-Ruby stain (BioRad).

### E2 Charging assay

E1-E2 thioester transfer assays to control for charging of the various E2s used throughout this study were performed as described for the HHARI autoubiquitination assays except that E3 was not added and the reaction time was 3 min.

### Western blot

Samples from autoubiquitination assays were resolved on SDS-PAGE and were transferred to PVDF (ThermoFisher) membranes using wet transfer method. The membranes were blocked with 5% non-fat milk, and incubated with primary and secondary antibodies sequentially with 3–5 washes for 10 min. Antibodies are as follows: UbcH7 (BostonBiochem A-640) used at a 1:1000 dilution, UbcH5b (ThermoFisher PA5-30968) used at a 1:2000 dilution, Ub (ThermoFisher PA 1–187) used at a 1:2000 dilution, and ECL-labeled anti-rabbit antibody (ThermoFisher NA934VS) used at a 1:10,000 dilution. Signal was developed with ECL substrates visualized by BioRad Geldoc.

### ITC

ITC experiments were performed on a nanoITC (TA instruments) at 25 °C in buffer containing 50 mM NaCl, 10 mM Na_2_HPO_4_, 2 mM KH_2_PO_4_ pH 7.0. Aliquots (2 μl each) of 200 μM UbcH7 proteins were injected into cell containing 20 μM HHARI full length. For UbcH5b proteins, 500 μM into 50 μM HHARI full length were used. Twenty measurements were made and the data were analyzed using NanoAnalyze (TA instruments).

### Size-exclusion analysis

Full-length HHARI and the indicated ubiquitin E2s were incubated in 20 mM Tris HCl pH 8.0, 50 mM NaCl, 2 mM βME at 4 °C overnight before going through Superose 12 10/300 GL size-exclusion column equilibrated in the same buffer. Fractions were analyzed on SDS-PAGE and visualized with Coomassie blue.

### Data availability

Atomic coordinates and structure factors are deposited in the RCSB with accession code 5TTE. The data that support the findings of this study are available from the corresponding author upon reasonable request.

## Electronic supplementary material

Supplementary Information
