# Supplementary material for: Structural insights into the mechanism and E2 specificity of the RBR E3 ubiquitin ligase HHARI
Source: Nat Commun. 2017 Aug 8;8:211. doi: 10.1038/s41467-017-00272-6 (PMC5548887; doi:10.1038/s41467-017-00272-6)
Supplement: Supplementary file 1 — Supplementary Information [file 41467_2017_272_MOESM1_ESM.pdf]

File name: Supplementary Information
Description: Supplementary figures and supplementary table.

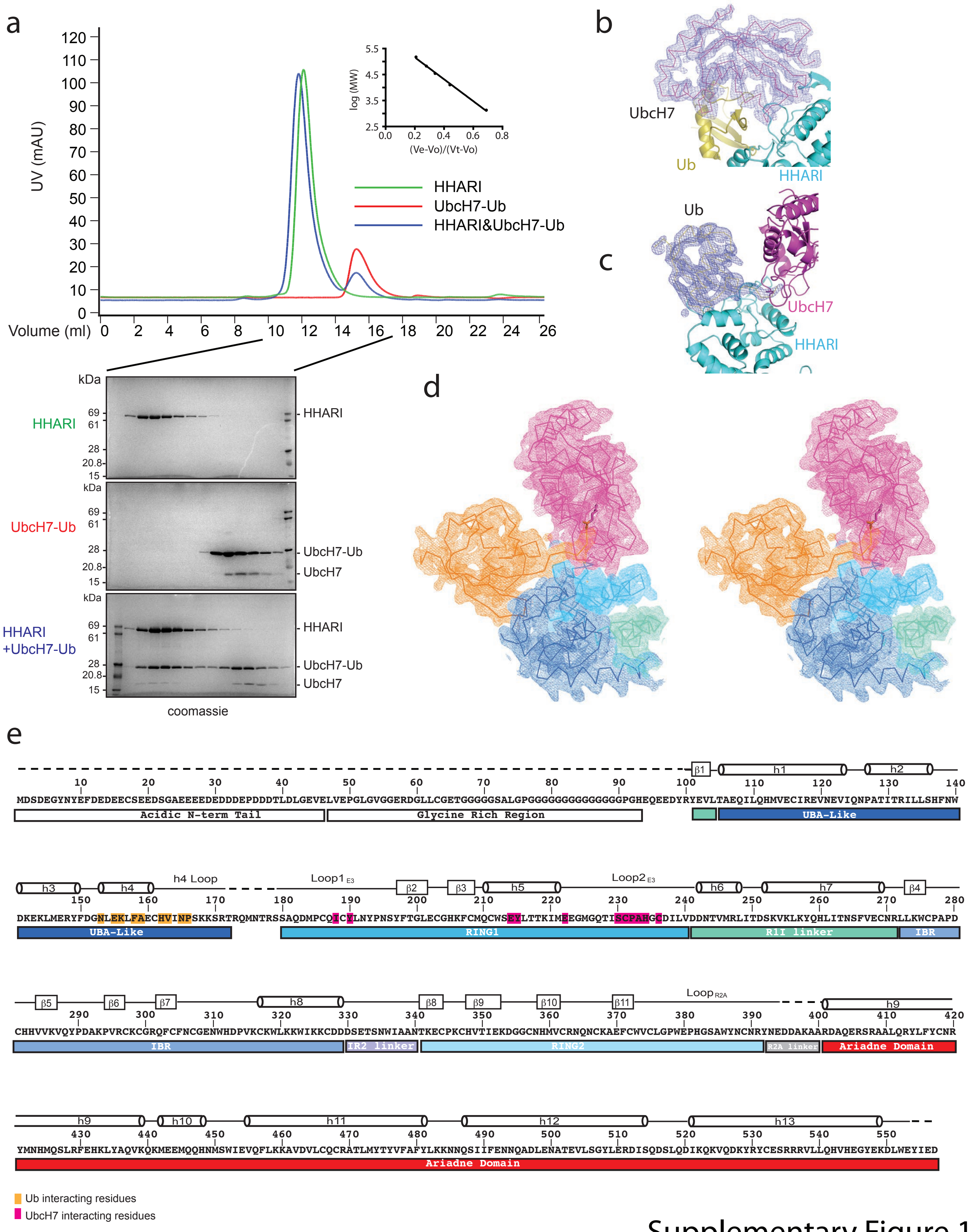

Supplementary Figure 1

Supplementary Figure 1 | HHARI/UbcH7-Ub purification, electron density, and secondary structure. | (**a**) HHARI alone, UbcH7-Ub alone, and HHARI mixed with a 1.5 fold molar excess of UbcH7-Ub were analyzed on a Superose 12 10/300 GL gel filtration column equilibrated in 20 mM Tris pH 8.0, 50 mM NaCl, and 2 mM β-mercaptoethanol. The indicated samples were analyzed by SDS-PAGE. (**b,c**) 2Fo-Fc electric density map contoured at 1.5 sigma for UbcH7 (**b**) and Ub(t) (**c**) in the HHARI/UbcH7-Ub structure. (**d**) A portion of the 2Fo-Fc electron density map focused on the interface between HHARI, UbcH7, and Ub, contoured at 1.5 sigma and presented as a wall-eye stereo image. Electron density maps and the accompanying Cα traces of the final protein model are colored as in **Fig. 1a**. (**e**) Sequence and structural features of HHARI. The secondary structure of HHARI is indicated above the sequence. Dashed lines indicate disordered regions of the structure. The domain organization of HHARI is indicated below the sequence. HHARI residues involved in contacts to UbcH7 and/or Ub are highlighted magenta and gold, respectively.

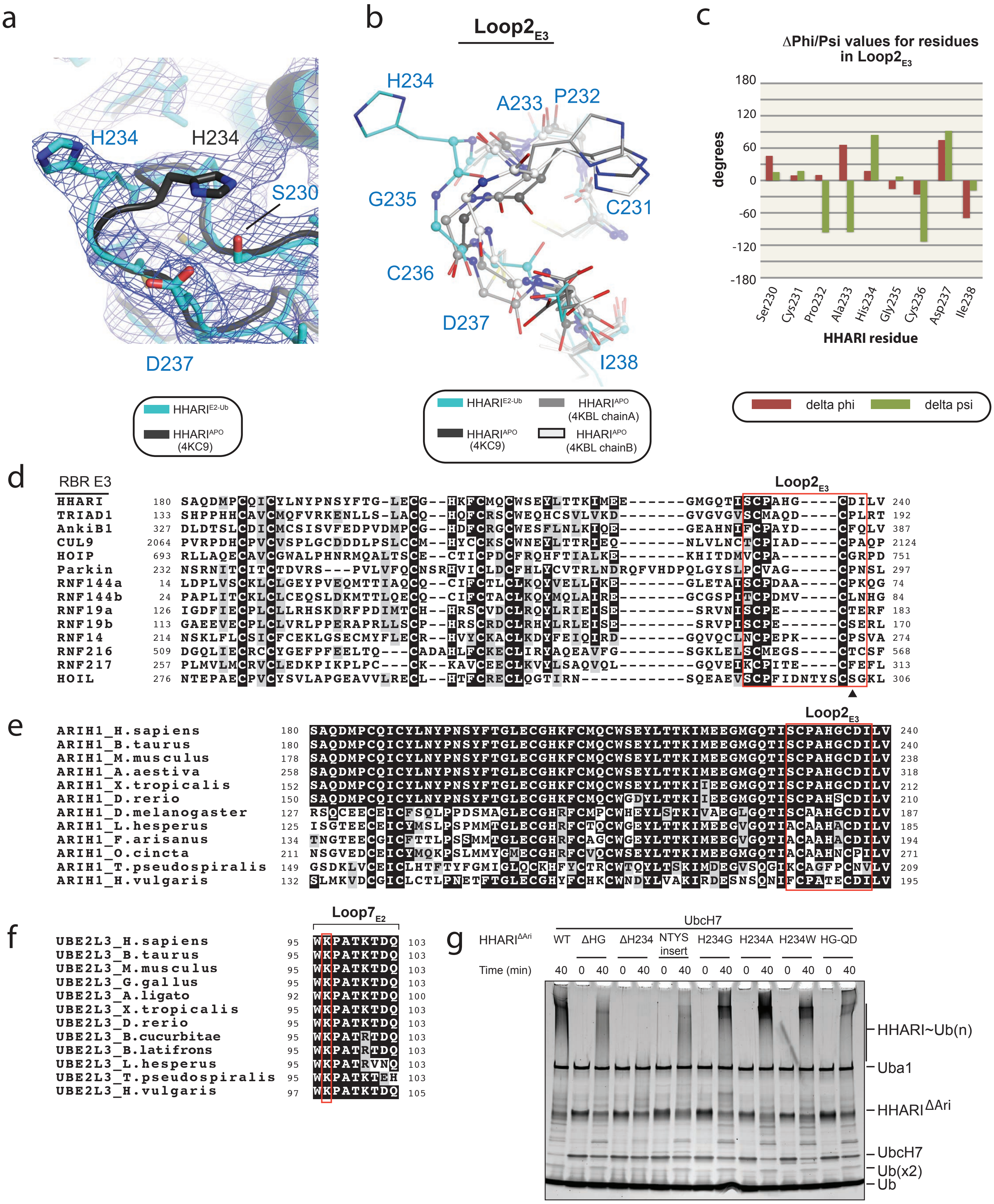

Supplementary Figure 2

Supplementary Figure 2 | Loop2$_{E3}$ of the HHARI RING1 domain undergoes a conformational change upon UbcH7-Ub binding. (**a**) 2Fo-Fc electric density map contoured at 1.5 sigma for the Loop2$_{E3}$ region of the HHARI RING1 domain in the HHARI/UbcH7-Ub structure. Loop2$_{E3}$ from a HHARI^{APO} structure (PDB: 4KC9) is shown to highlight the conformational change that occurs upon UbcH7-Ub binding. (**b**) The RING1 domains of HHARI from the HHARI/UbcH7-Ub structure and the three HHARI^{APO} structures (PDBs: 4KC9 and both copies of 4KBL) were superimposed. Loop2$_{E3}$ from the structures are shown as sticks in the same orientation as panel a. (**c**) Plot of the change in phi/psi angles for residues in Loop2$_{E3}$ of the of the RING1 domain in HHARI/UbcH7-Ub versus HHARI^{APO} structure (PDB: 4KC9). (**d**) Sequence alignment of the RING1 domain of the fourteen human RBR E3 ligases. Highly conserved residues are shaded black, moderately conserved residues are shaded gray. The Loop2$_{E3}$ region is boxed in red to highlight variability of the length of the Loop2$_{E3}$ insertion across the different RBR E3s. The 'linchpin' arginine residue of canonical RING E3s that is involved in stabilization of the closed E2~Ub conformation is indicated with a black triangle. (**e**) Sequence alignment of the HHARI RING1 domain across species, residues shaded as in **d**. The Loop2$_{E3}$ region is boxed in red to highlight the same length of the Loop2$_{E3}$ insertion across the different HHARI species. (**f**) Sequence alignment of the Loop7$_{E2}$ of UBE2L3 across various species, residues shaded as in **d**. The E2 position (corresponding to Lys96 of human UBE2L3) we demonstrate is important for HHARI specificity is highlighted with a red box. (**g**) Structure-function analysis of the HHARI RING2 Loop2$_{E3}$ insertion. WT and mutant proteins were utilized in HHARI autoubiquitination assays for the indicated time points, as described in the Methods.

a

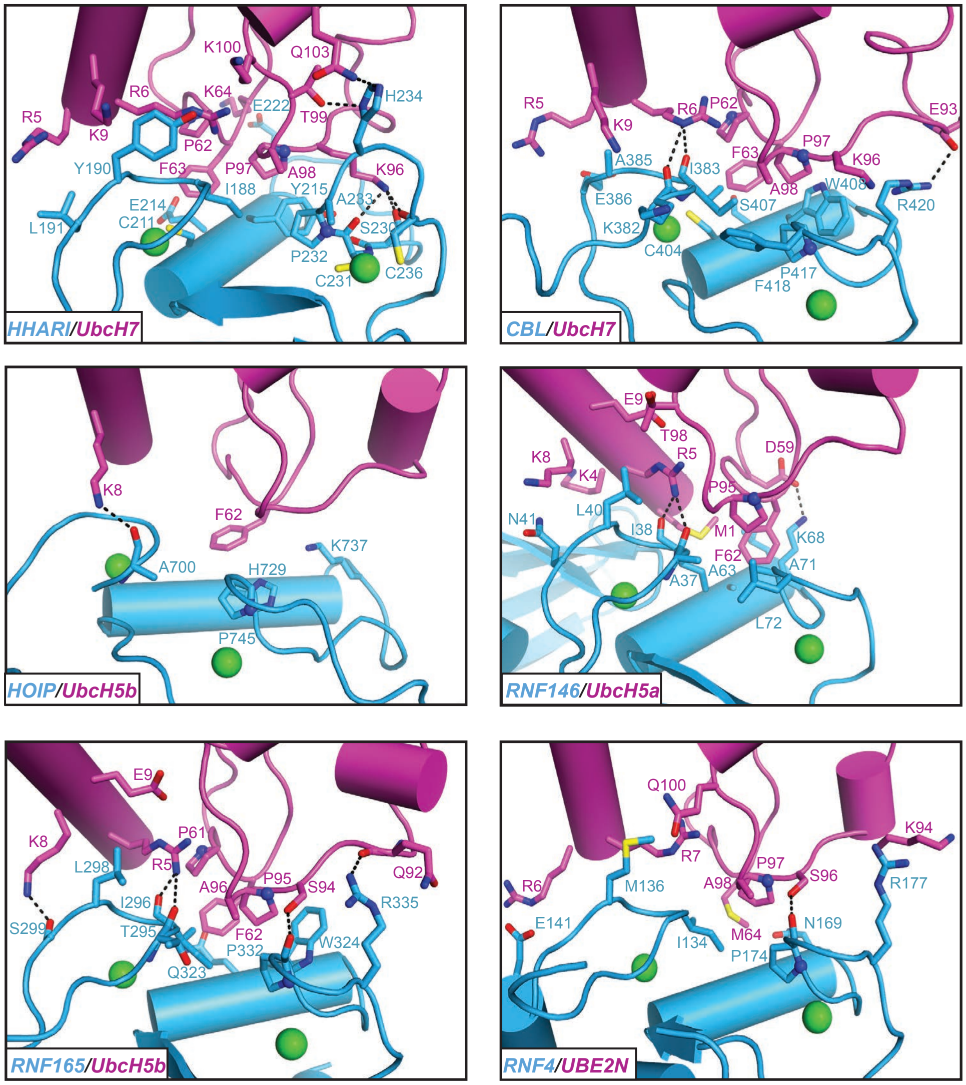

d

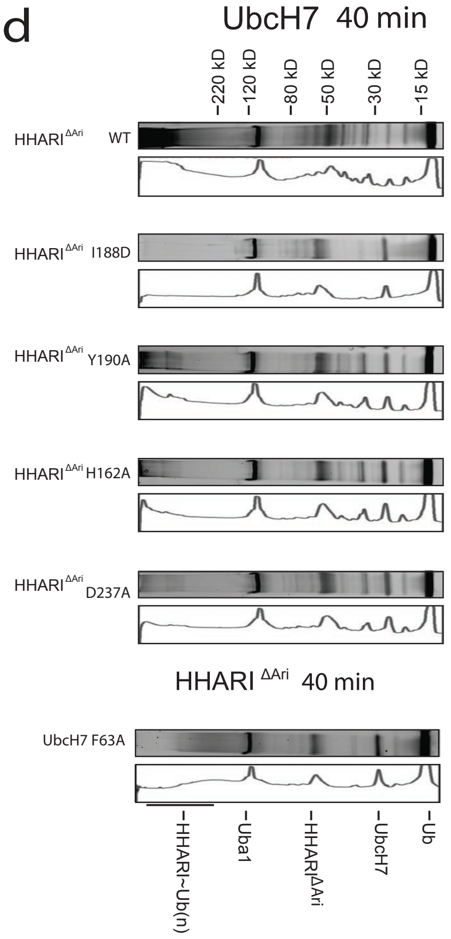

b

E3	E2		h1			Loop4$_{E2}$			★ … Loop7$_{E2}$	
HHARI	UbcH7	1	MAASRRLMKELEEIRK	16	60	EYPFKPP	66	85	VCLPVISAEN------------WKPATKTDQVIQ	106
c-CBL	UbcH7	1	MAASRRLMKELEEIRK	16	60	EYPFKPP	66	85	VCLPVISAEN------------WKPATKTDQVIQ	106
RNF146	UbcH5a	1	MALKRIQKELSDLQR	15	59	DYPFKPP	65	84	ICLDILRSQ-------------WSPALTVSKVLL	104
RING1b	UbcH5a	1	MALKRIQKELSDLQR	15	59	DYPFKPP	65	84	ICLDILRSQ-------------WSPALTVSKVLL	104
RNF25	UbcH5b	1	MALKRIHKELNDLAR	15	59	DYPFKPP	65	84	ICLDILRSQ-------------WSPALTISKVLL	104
RNF165	UbcH5b	1	MALKRIHKELNDLAR	15	59	DYPFKPP	65	84	ICLDILRSQ-------------WSPALTISKVLL	104
RNF38	UbcH5b	1	MALKRIHKELNDLAR	15	59	DYPFKPP	65	84	ICLDILRSQ-------------WSPALTISKVLL	104
HOIP	UbcH5b	1	MALKRINKELSDLAR	15	59	DYPFKPP	65	84	ICLDILRSQ-------------WSPALTISKVLL	104
NOT4	ScUbc4	1	MSSSKRIAKELSDLER	16	60	DYPFKPP	66	85	ICLDILKDQ-------------WSPALTLSKVLL	105
FANCL	hUBE2T	1	MQRASRLKRELHMLAT	16	60	RYPFEPP	66	85	ICLDVLKLPPKG---------AWRPSLNIATVLT	109
RNF4	hUBE2N	2	AGLPRRIIKETQRLLA	17	61	EYPMAAP	67	86	ICLDILKD-------------KWSPALQIRTVLL	106
GP78	hUBE2G2	3	GTALKRLMAEYKQLTL	18	63	DYPLSPP	69	88	VCISILHAPGDDPMGYESSAERWSPVQSVEKILL	121
APC11	UbcH10	29	GPVGKRLQQELMTLMM	44	88	GYPYNAP	94	113	ICLDILKE-------------KWSALYDVRTILL	139

c

E2	E3		Loop1$_{E3}$ … β2 … β3 … H5			Loop2$_{E3}$		Z-score	rmsd
UbcH7	HHARI	180	SAQDMPCQICYLNYPN-----SYFTGLECGHKFCMQCWSEYLTTKIM	221	222	EEGMGQTISCPAHGCDILV	240	-	-
UbcH7	c-CBL	375	GSTFQLCKICAEND-------KDVKIEPCGHLMCTSCLTSWQESEG-	413	414	-------QGCPF--CRCEI	423	4.3	2.7
UbcH5a	RNF146	30	SLTVPECAICLQTC-------VHPVSLPCKHVFCYLCVKGASWLG--	67	68	-------KRCAL--CRQEI	77	4.8	1.9
UbcH5a	RING1b	45	LHSELMCPICLDMLK------NTMTTKECLHRFCADCIITALRSG--	83	84	------NKECPT--CRKKL	94	3.9	3.1
UbcH5b	RNF25	129	NIPHGQCVICLYGFQEK----EAFTKTPCYHYFHCHCLARAIQHMEQ	171	188	TKQKAVGVQCPV--CREPL	205	5.6	2.3
UbcH5b	RNF165	288	SDTDEKCTICLSMLEDG----EDVRRLPCMHLFHQLCVDQWLAMS--	328	329	-------KKCPI--CRVDI	338	4.4	2.4
UbcH5b	RNF38	457	QSEQTLCVVCMCDFESR----QLLRVLPCNHEFHAKCVDKWLKAN--	497	498	-------RTCPI--CRADA	507	4.0	2.0
UbcH5b	HOIP	693	RLLAQECAVCGWALPHN----RMQALTSCECTICPDCFRQHFTIALK	735	736	-EKHITDMVCPA--CGRPD	751	3.3	2.5
ScUbc4	NOT4	27	EDEEDYCPLCIEPMDITD---KNFFPCPCGYQICQFCYNNIRQN---	67	68	---PELNGRCPA--CRRKY	81	4.9	1.9
hUBE2T	FANCL	301	SDFTMDCGICYAYQLDGTIPDQVCDNSQCGQPFHQICLYEWLRGLLT	347	350	QSFNIIFGECPY--CSKPI	366	3.7	2.3
hUBE2N	RNF4	126	PSGTVSCPICMDGYSEIVQNGRLIVSTECGHVFCSQCLRDSLKNA--	170	171	-------NTCPT--CRKKI	180	4.7	3.0
hUBE2G2	GP78	335	AVNNDDCAICWDSM-------QAARKLPCGHLFHNSCLRSWLEQD--	372	373	-------TSCPT--CRMSL	382	n/a	n/a
UbcH10	APC11	28	MAFNGCCPDCKVPGDD-----CPLVWGQCSHCFHMHCILKWLHAQQV	69	70	------QQHCPM--CRQEW	80	n/a	n/a

Supplementary Figure 3

Supplementary Figure 3 | Comparison of the HHARI RING1/UbcH7 interface to canonical RING E3/E2 interfaces (**a**) Selected RING E3/E2 interfaces are shown as cartoon representations in the same orientation as HHARI/UbcH7 with residues involved in intermolecular contacts shown as sticks. Dashed lines indicate hydrogen bonds. Zinc atoms are shown as green spheres. (**b**) Sequence alignment of E2s from complexes with the indicated RING E3s highlighting E2 regions involved in intermolecular contacts to the RING domain. E2 residues involved in contacts to the indicated E3 are shaded light blue. The E2 catalytic cysteine is indicated with a red star. (**c**) Sequence alignment of RING E3s from complexes with the indicated E2s highlighting E3 regions involved in intermolecular contacts to the E2. E3 residues involved in contacts to the indicated E2 are shaded light magenta. Residues involved in coordination of zinc atoms are indicated with a black star below the alignment. The 'linchpin' arginine residue conserved in canonical RING E3s is indicated with a black triangle below the alignment. Atypical residues involved in zinc atom coordination (Cys324 of FANCL and Cys50 of NOT4) are highlighted with a black box in the alignment. Z-scores and rmsd values calculated from superimpositions of the indicated RING domain structure to the HHARI RING1 domain structure are listed to the right of the alignment. (**d**) The 40 minute time points from the HHARI autoubiquitination assays probing the RING1/UbcH7-Ub interface displayed in Figure 4d are presented as a waterfall plot. The results were generated with ImageJ.

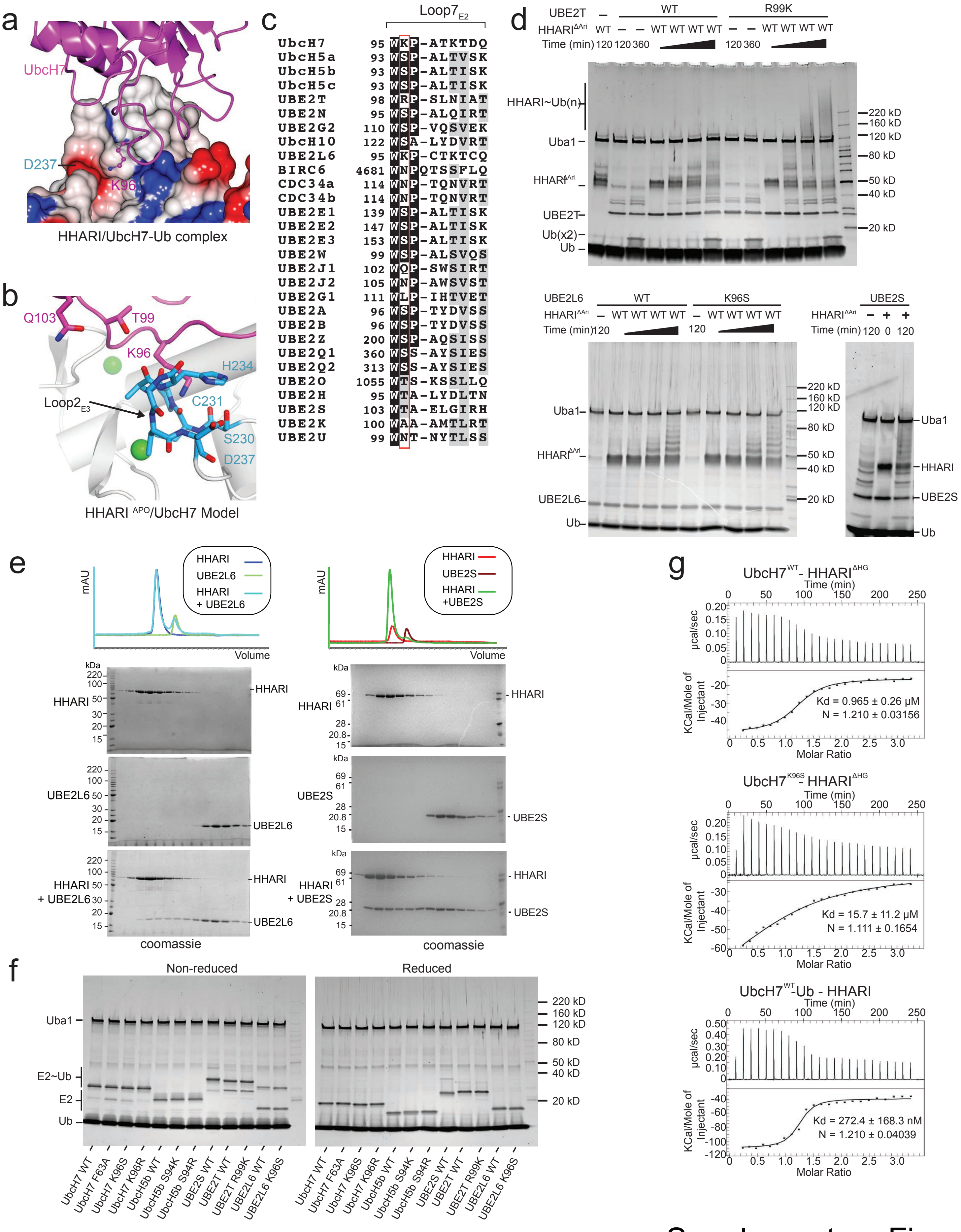

Supplementary Figure 4

Supplementary Figure 4 | The Loop2$_{E3}$ conformational change of the HHARI RING1 domain unmasks a critical UbcH7 binding site. (**a**) Lys96 of UbcH7 projects towards an acidic surface on the HHARI RING1 domain that is unmasked by the Loop2$_{E3}$ conformational change. The HHARI RING1 domain is shown as a surface electrostatic representation and UbcH7 is shown as a cartoon with the Lys96 side chain shown as sticks. Ub(t) is not shown for clarity. (**b**) A HHARI^{APO}/UbcH7 model was generated by superimposing UbcH7 onto the RING1 domain of HHARI^{APO}. This model shows that Lys96 of UbcH7 would severely clash with HHARI Loop2$_{E3}$ in the apo conformation. (**c**) A sequence alignment of the Loop7$_{E2}$ region of active human ubiquitin E2s. Highly conserved residues are colored black, moderately conserved residues are colored gray, and the E2 position (corresponding to Lys96 of UbcH7) we demonstrate is important for HHARI specificity is highlighted with a red box. (**d**) WT and mutant UBE2T were utilized in HHARI autoubiquitination assays for the indicated time points, as described in the Methods (**top**). UBE2L6 WT, UBE2L6 K96S, and UBE2S subjected to HHARI autoubiquitination assays (**bottom**). (**e**) Analytical size exclusion analysis of FL HHARI and UBE2L6 (left) and FL HHARI and UBE2S (right). (**f**) E2 charging controls for the various E2s used throughout this study. The E1-E2 thioester transfer assays were performed as described in the Methods. (**g**) Isothermal titration calorimetry data for interactions between HHARI full-length ΔHG and UbcH7^{WT} or UbcH7^{K96S}, and between HHARI full-length and UbcH7-Ub conjugate. Upper panels show raw power data and lower panels show fits of the data to standard binding equations using NanoAnalyze software (TA instruments).

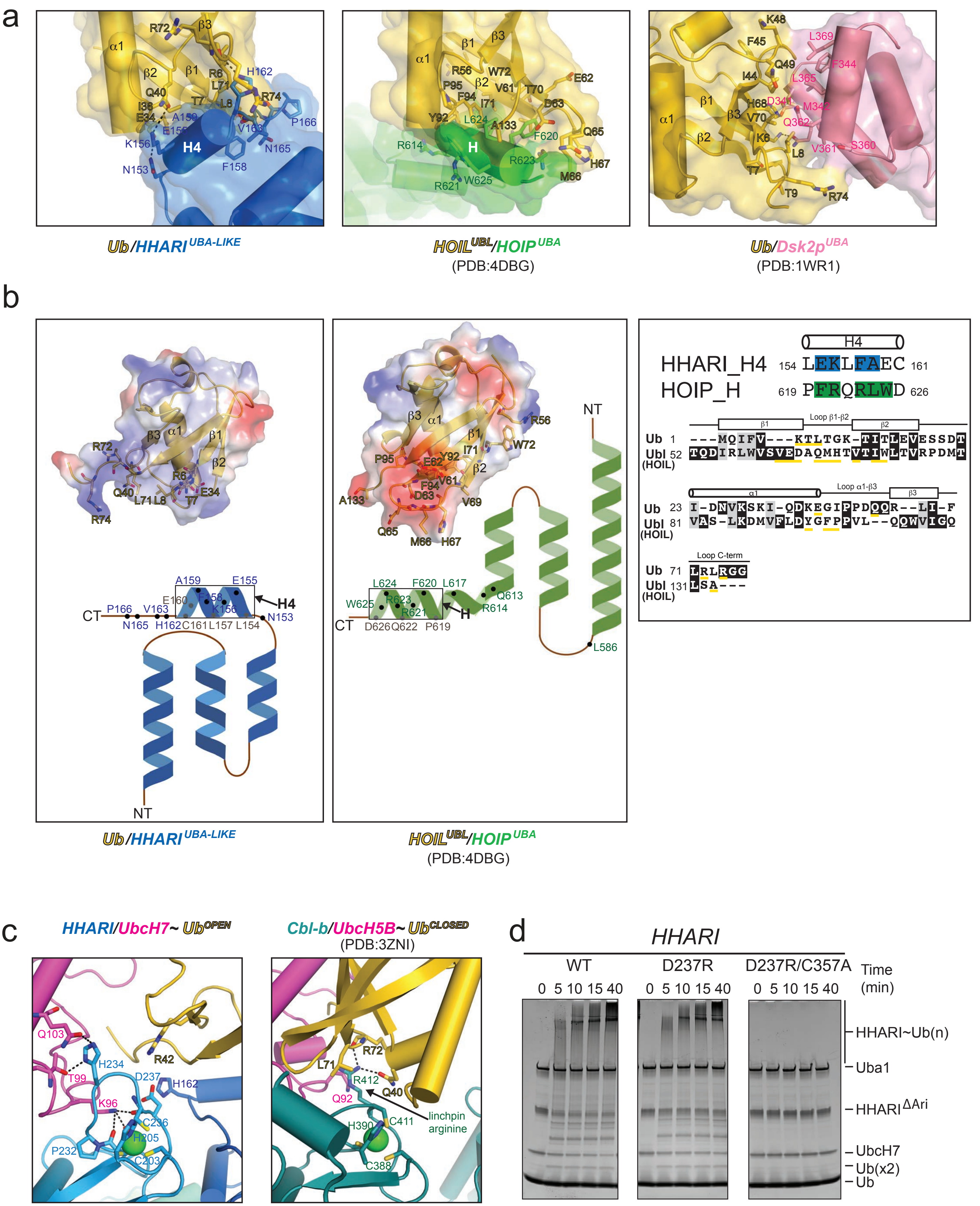

Supplementary Figure 5

Supplementary Figure 5 | The HHARI$^{UBA\text{-}like}$/Ub interaction is distinct from canonical UBA/Ub interactions and most closely resembles the HOIP^{UBA}/HOIL^{UBL} interface. (**a**) The HHARI$^{UBA\text{-}like}$/Ub (*left*), HOIP^{UBA}/HOIL^{UBL} (*center*), and Dsk2p^{UBA}/Ub (*right*) structures are shown as cartoon representations in the same orientation with residues involved in intermolecular contacts shown as sticks. (**b**) The HHARI$^{UBA\text{-}like}$/Ub (left) and HOIP^{UBA}/HOIL^{UBL} (center) intermolecular contacts are shown in the same orientation. Transparent eletrostatic representation of the interface of Ub with HHARI$^{UBA\text{-}like}$ (top left) and transparent eletrostatic representation of the interface of HOIL^{UBL} with HOIP^{UBA} (top center). Major contacts are shown as sticks. Schematic representation of the HHARI$^{UBA\text{-}like}$ contacts with Ub (bottom left) and schematic representation of the HOIP^{UBA} contacts with HOIL^{UBL} (bottom center). Black spots indicate the interacting residues, and gray spots indicate the non-interacting residues. The sequence alignment between HHARI_H4 and HOIP_H is shown on the top right, and the contacts residues are colored blue and green, respectively. The sequence alignment between Ub and HOIL^{UBL} is shown on the bottom right, and the contacts residues are outlined in yellow. Highly conserved residues are shaded black, moderately conserved residues are shaded gray.

(**c**) The 'linchpin' arginine residue of canonical RING E3s plays a crucial role in catalysis by engaging in interactions with Ub that stabilize the closed E2~Ub thioester intermediate conformation. A representative set of interactions that the linchpin arginine residue engages in is observed in the pTyr363-Cbl-b/UbcH5b-Ub structure (PDB: 3ZNI) which is shown in the **right** panel as cartoon representation with selected residues involved in intermolecular interactions shown as sticks. The linchpin arginine is not conserved in any of the RBR E3s (also see **Supplementary Fig. 2d**). The HHARI/UbcH7-Ub structure is shown in the same orientation in the **left** panel to allow for comparison. The HHARI residue corresponding to the linchpin residues of canonical RING E3s is Asp237. (**d**) Time course autoubiquitination assay of HHARI WT, D237R, and D237R/C357A.

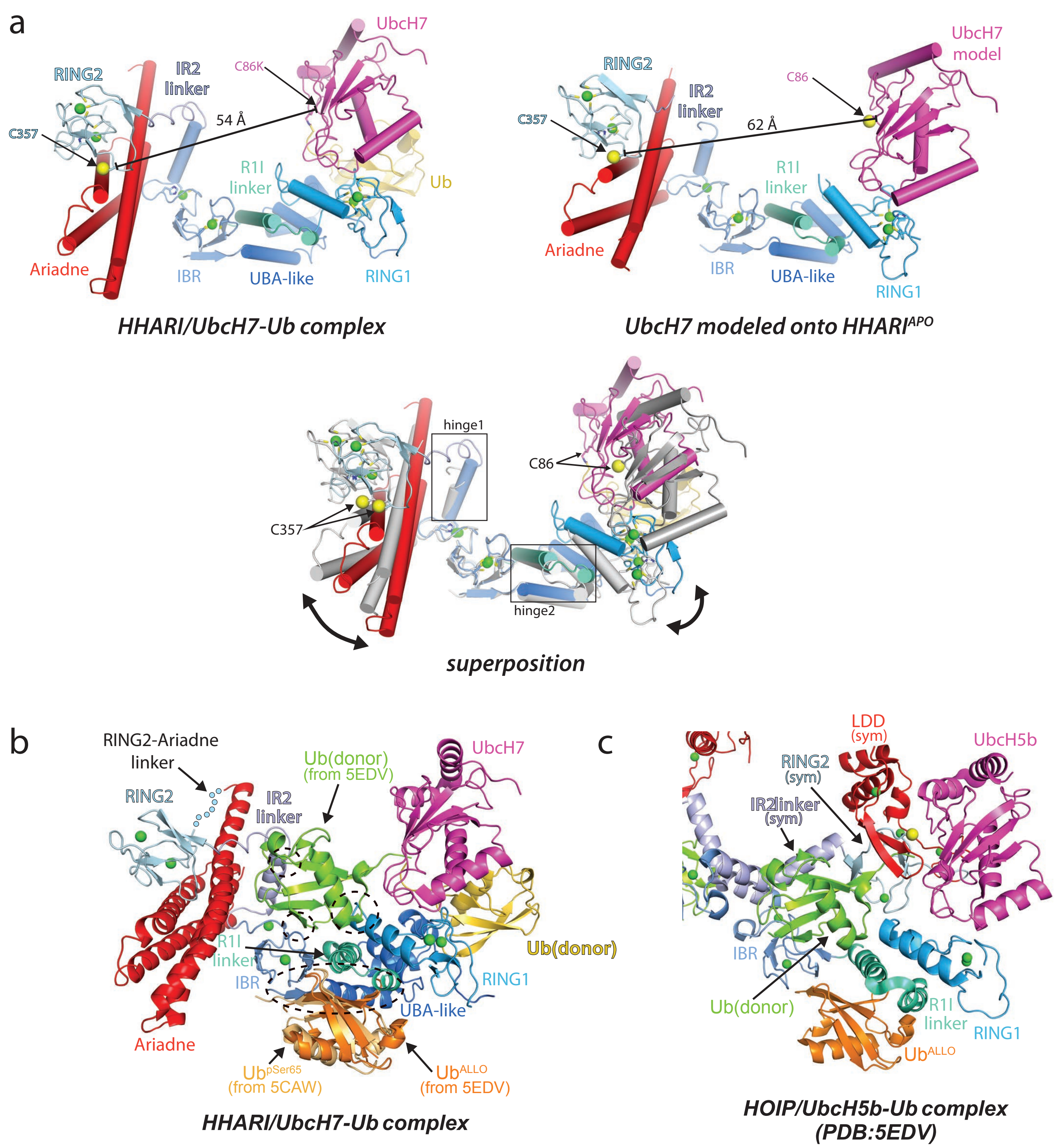

Supplementary Figure 6

Supplementary Figure 6 | Comparison of the HHARI/UbcH7-Ub structure to other RBR E3 structures. (a) The HHARI/UbcH7-Ub structure is shown in the top panel. A model of an $HHARI^{APO}$/UbcH7 complex was created by superimposing RING1/UbcH7 from the HHARI/UbcH7-Ub structure onto RING1 of an apo HHARI structure (PDB 4KC9) and is presented in the bottom panel. The IBR domains of the HHARI/UbcH7-Ub structure (colored as in Fig. 1a) and the $HHARI^{APO}$/UbcH7 model (colored gray) were superimposed and presented in the middle panel. The distances between the UbcH7 and HHARI catalytic cysteine residues are indicated with arrows. Hinge regions where conformational changes are likely to occur in order to bring the UbcH7 and HHARI active sites together during thioester transfer of Ub are highlighted in black boxes in the middle panel. The domain rotations that result from conformational changes in the Hinge 1 and 2 regions are indicated by double-headed black arrows. (**b**) The HHARI/UbcH7-Ub structure is shown as a cartoon representation. Ub(donor) and Ub^{ALLO} from the HOIP/UbcH5b-Ub structure (PDB: 5EDV) and pSer65-Ubiquitin (pUb) from the Parkin/pUb structure (PDB:5CAW) were modeled onto HHARI by superimposing the IBR domains of the RBR E3s. Steric clashes between HHARI and Ub(donor) and Ub^{ALLO} based on the HOIP/UbcH5b-Ub structure and pUb based on the Parkin/pUb structure are indicated with dashed ovals. (**c**) The HOIP/UbcH5b-Ub structure (PDB: 5EDV) is shown as a cartoon representation similar to HHARI in panel **b** in order to allow for comparison.

Supplementary Table 1. Primers used to generate all constructs used in this study

HHARI primers	Primer sequences(listed in 5' to 3' orientation)
HHARI FL	FORWARD: GGTGGATCCATGGACTCGGACGAGGGCTACAACTAC<br>REVERSE: CCGGGCGGCCGCTCAGTCCTCAATGTACTCCCACAGATC
HHARI ΔAri	FORWARD: GGATGATGCAAAGGCAGCATAAGATGCACAGGAGCGATCTAGG<br>REVERSE: CCTAGATCGCTCCTGTGCATCTTATGCTGCCTTTGCATCATCC
C357A	FORWARD: TTGAGAAGGATGGTGGTGCTAATCACATGGTCTGT<br>REVERSE: ACAGACCATGTGATTAGCACCACCATCCTTCTCAA
I188D	FORWARD: GATATGCCTTGTCAGGACTGCTACTTGAACTACCCT<br>REVERSE: AGGGTAGTTCAAGTAGCAGTCCTGACAAGGCATATC
Y190A	FORWARD: GCCTTGTCAGATCTGCGCCTTGAACTACCCTAACT<br>REVERSE: AGTTAGGGTAGTTCAAGGCGCAGATCTGACAAGGC
H162A	FORWARD: CTCTTTGCTGAGTGTGCTGTAATTAATCCAAGT<br>REVERSE: ACTTGGATTAATTACAGCACACTCAGCAAAGAG
D237A	FORWARD: CCTGCTCATGGTTGTGCTATCTTAGTGGATGAC<br>REVERSE: GTCATCCACTAAGATAGCACAACCATGAGCAGG
K156A	FORWARD: GATGGAAACCTGGAGGCGCTCTTTGCTGAGTGTCATG<br>REVERSE: CATGACACTCAGCAAAGAGCGCCTCCAGGTTTCCATC
A159D	FORWARD: AACCTGGAGAAGCTCTTTGATGAGTGTCATGTAATTAATC<br>REVERSE: GATTAATTACATGACACTCATCAAAGAGCTTCTCCAGGTT
I164W	FORWARD: CTTTGCTGAGTGTCATGTATGGAATCCAAGTAAAAAGTCT<br>REVERSE: AGACTTTTTACTTGGATTCCATACATGACACTCAGCAAAG
A159D/I164W	FORWARD: AACCTGGAGAAGCTCTTTGATGAGTGTCATGTATGGAATC<br>REVERSE: GATTCCATACATGACACTCATCAAAGAGCTTCTCCAGGTT
T333P/S334P	FORWARD: GATGATGACAGTGAACCCCCCAATTGGATTGCAGCCAAC<br>REVERSE: GTTGGCTGCAATCCAATTGGGGGGTTCACTGTCATCATC
T333P/S334P/ A338P/A339P	FORWARD: GAACCCCCCAATTGGATTCCACCCAACACAAAGGAATG<br>REVERSE: CATTCCTTTGTGTTGGGTGGAATCCAATTGGGGGGTTC
Δ333-339	FORWARD: TTCACTGTCATCATCACACTTTTTAATCCATTTC<br>REVERSE: AACACAAAGGAATGTCCCAAATGCCATGTC
Δ322-339	FORWARD: TTCACTGTCATCATCACACTTTTTAATCCATTTC<br>REVERSE: CTTTAACCACTTACATTTAACAGGATCATGCC
Δ234-235	FORWARD: AGCAGGACACGAAATAGTCTGACCCATGCCTTCT<br>REVERSE: TGTGATATCTTAGTGGATGACAACACAGTTATG
Δ234	FORWARD: GACTATTTCGTGTCCTGCTGGTTGTGATATCTTAGTG<br>REVERSE: CACTAAGATATCACAACCAGCAGGACACGAAATAGTC
NTYS insert after G235	FORWARD: GGTGTTACCATGAGCAGGACACGAAATAGTCTGAC<br>REVERSE: TACAGCTGTGATATCTTAGTGGATGACAACACAGTTATGC
H234G	FORWARD: GACTATTTCGTGTCCTGCTGGTGGTTGTGATATCTTAGTG<br>REVERSE: CACTAAGATATCACAACCACCAGCAGGACACGAAATAGTC
H234A	FORWARD: CTATTTCGTGTCCTGCTGCTGGTTGTGATATCTTA<br>REVERSE: TAAGATATCACAACCAGCAGCAGGACACGAAATAG
H234W	FORWARD: CTATTTCGTGTCCTGCTTGGGGTTGTGATATCTTA<br>REVERSE: TAAGATATCACAACCCCAAGCAGGACACGAAATAG

H234Q/G235D	FORWARD: CTATTTCGTGTCCTGCTCAAGATTGTGATATCTTAGTGG<br>REVERSE: CCACTAAGATATCACAATCTTGAGCAGGACACGAAATAG
D237R	FORWARD: CCTGCTCATGGTTGTCGAATCTTAGTGGATGACA<br>REVERSE: TGTCATCCACTAAGATTCGACAACCATGAGCAGG
**E2 primers**	
UbcH7 WT	FORWARD: GGTGGTCCATGGCGGCCAGCAGGAGGCTGATGAA<br>REVERSE: CCGGAAGCTTGTCCACAGGTCGCTTTTCCCCATATTTC
UbcH7 F63A	FORWARD: TCCAGCAGAGTACCCAGCCAAACCACCGAAG<br>REVERSE: CTTCGGTGGTTTGGCTGGGTACTCTGCTGGA
UbcH7 K96S	FORWARD: AGTGCCGAAAACTGGAGCCCAGCAACCAAAACCGAC<br>REVERSE: GTCGGTTTTGGTTGCTGGGCTCCAGTTTTCGGCACT
UbcH7 K96R	FORWARD: GTGCCGAAAACTGGCGGCCAGCAACCAAAACCGA<br>REVERSE: TCGGTTTTGGTTGCTGGCCGCCAGTTTTCGGCAC
UbcH7 C86K	FORWARD: GAAAAGGGGCAGGTCAAACTGCCAGTAATTAG<br>REVERSE: CTAATTACTGGCAGTTTGACCTGCCCCTTTTC
UbcH5b WT	FORWARD: GGTGGTCATATGGCTCTGAAGAGAATCCACAAGGAATTG<br>REVERSE: CCGGAAGCTTCATCGCATACTTCTGAGTCCATTCCCG
UbcH5b S94K	FORWARD: TCTACGATCACAGTGGAAGCCAGCACTAACTATTTC<br>REVERSE: GAAATAGTTAGTGCTGGCTTCCACTGTGATCGTAGA
UbcH5b S94R	FORWARD: TCTACGATCACAGTGGCGACCAGCACTAACTATTTC<br>REVERSE: GAAATAGTTAGTGCTGGTCGCCACTGTGATCGTAGA
UBE2S WT	FORWARD: GGTGGTACATGTCTATGAACTCCAACGTGGAGAACCTACCCC<br>REVERSE: CCGGCTCGAGCTACAGCCGCCGCAGCGCCC
UBE2T WT	FORWARD: GGTGGTCATATGCAGAGAGCTTCACGTCTGAAGAGAG<br>REVERSE: CCGGAAGCTTAACATCAGGATGAAATTTCTTTTCTATGCCTAC
UBE2T R99K	FORWARD: CCACCAAAAGGTGCTTGGAAACCATCCCTCAACATCG<br>REVERSE: CGATGTTGAGGGATGGTTTCCAAGCACCTTTTGGTGG
UBE2L6 WT	FORWARD: GGACATATGATGGCGAGCATGCGAGTGGTGAAGG<br>REVERSE: GCCAAGCTTGGAGGGCCGGTCCACTCCGAATCGG
UBE2L6 K96S	FORWARD: CAGCAGTGAGAACTGGTCGCCTTGCACCAAGAC<br>REVERSE: GTCTTGGTGCAAGGCGACCAGTTCTCACTGCTG
